# Occupational Therapy Within Integrated Community Care Systems: A Systematic Review and Narrative Synthesis of Policy and System-Level Evidence

**DOI:** 10.3390/healthcare14193222

**Published:** 2026-09-30

**Authors:** Su-Min Cha

**Affiliations:** Department of Occupational Therapy, Kyungnam University, Changwon 51767, Republic of Korea; csm1206@kyungnam.ac.kr; Tel.: +82-55-249-6479

**Keywords:** occupational therapy, integrated care, community care, health policy, financing, referral, service accessibility, workforce, implementation

## Abstract

Background/Objectives: Population aging and rising long-term-care demand require community-based systems that support function and participation. Occupational therapy (OT) is provided across community-care settings, yet its policy- and system-level position remains inconsistently defined. This review examined how OT is positioned, financed, accessed, coordinated, staffed, and implemented within integrated community care. Methods: PubMed, CINAHL, Scopus, ProQuest Central, and APA PsycINFO were searched for English-language peer-reviewed literature published from 1 January 2010 to 29 July 2026. Two reviewers independently conducted study selection, data extraction, and design-matched critical appraisal. GRADE-CERQual and GRADE were applied to eligible qualitative and quantitative findings, respectively. The review synthesized peer-reviewed evidence describing policy- and system-level arrangements rather than systematically reviewing primary policy documents themselves. Results: Of 15,402 records, 5131 duplicates were removed, 10,271 screened, and 38 reports assessed in full text; 21 reports representing 20 unique studies/datasets were included. Framework synthesis refined the initial deductive categories into five higher-order domains: institutional positioning; financing/coverage; referral/coordination; service-delivery roles/infrastructure; and workforce/implementation support, with accessibility, equity, and user participation as cross-cutting themes. CERQual confidence was moderate for role clarity/team understanding, organizational integration supports, and service context/workforce capacity, and low for reimbursement/billing constraints, referral-prioritization/fairness judgments, and service-redesign tensions. Waiting-time change, waiting-list association, and OT integration feasibility had very low GRADE certainty. Conclusions: OT integration reflected interrelated policy-system arrangements rather than a validated model. Downstream system effects remain insufficiently established.

## 1. Introduction

Population aging and the rising prevalence of chronic conditions and multimorbidity are heightening the need for health systems to address sustained, complex care needs [1]. Across OECD countries, chronic conditions become increasingly common with age, and multimorbidity is particularly prevalent among older adults [1]. The WHO framework on integrated, people-centered health services identifies fragmentation and weak coordination across services as system barriers to continuity and person-centered care [2]. The subsequent WHO report on strengthening integrated, people-centered health services further emphasizes coordinated, system-level organization rather than isolated episodes of care [3]. These developments have made integrated, person-centered care a major policy priority, with a focus on reorganizing and coordinating services rather than simply adding isolated services [2,3].

Integrated care has been conceptualized in several complementary ways [4,5,6]. Kodner and Spreeuwenberg described it as a coherent set of methods and models designed to foster connectivity, alignment, and collaboration within and across levels of care [4]. Valentijn et al. organized integration across system, organizational, professional, and clinical levels in the Rainbow Model of Integrated Care [5]. Goodwin emphasized that integrated care is context-dependent and should not be reduced to a single organizational template [6]. Evidence syntheses likewise show that outcomes depend on how integration is configured [7,8]. Baxter et al. reported heterogeneous effects across integrated-care programs and outcomes, with some improvements in service quality, patient satisfaction, and access, but no uniformly effective model [7]. In community-based multidisciplinary care involving general practitioners, Hayes et al. found long-term improvements in functional status, hospitalization, and patient satisfaction, while evidence for several other outcomes was non-significant and generally of low or very low certainty [8]. Taken together, these conceptual and empirical sources support the understanding that integrated care is the organization of roles, pathways, financing, coordination, and governance mechanisms, rather than simply the addition of community programs [4,5,6,7,8].

For this review, policy- and system-level evidence was defined in relation to health-system and integrated-care concepts rather than as a synonym for clinical effectiveness [2,5,6]. The WHO framework identifies governance, coordination, service-delivery organization, and enabling system conditions as central to integrated people-centered care [2]. Valentijn et al. distinguish system and organizational integration from professional and clinical integration [5], while Goodwin stresses that the form of integration depends on local institutional and service contexts [6]. On this basis, policy- and system-level evidence in the present review was operationalized as peer-reviewed evidence describing the rules, institutional arrangements, organizational mechanisms, and implementation conditions that shape or constrain the institutional positioning and delivery of occupational therapy within community-based care systems [2,5,6]. The scope covered macro-level policy and financing arrangements, meso-level service-system arrangements, and cross-cutting issues of accessibility, equity, and user participation, consistent with the multi-level and people-centered framing of integrated care [2,5]. Individual-level clinical outcomes or intervention effectiveness were therefore not considered policy- or system-level evidence in themselves and were included in this interpretation only when explicitly linked to such policy or system arrangements [2,5,6].

Within this policy-and-system scope, rehabilitation has attracted growing international attention as a health-system priority [9]. The WHO Rehabilitation 2030 initiative calls for strengthening rehabilitation through governance, workforce development, financing mechanisms, and integrated service delivery across levels of care [9]. Global estimates from the Global Burden of Disease Study 2019 indicate that approximately 2.41 billion people had conditions that would benefit from rehabilitation in 2019 [10]. Occupational therapy services in primary care have been described as supporting function, daily living, participation, and self-management across diverse populations [11]. Freeman et al. [12] documented wide international variation in the organization, financing, workforce supply, and training needs of occupational therapy in primary care, highlighting substantial differences in how occupational therapy is positioned and supported within primary care systems. Complementing this system-level evidence, Cumming et al. [13] reported benefits of occupational therapist-led home hazard assessment and modification for fall prevention, particularly among older adults with a history of falls. Pighills et al. [14] further examined the effectiveness of occupational therapist-led environmental assessment and modification in preventing falls, while Stark et al. [15] provided more recent evidence that occupational therapist-led home hazard removal can reduce fall rates among community-dwelling older adults. In contrast, Legg et al. [16] found no eligible comparative studies establishing the effectiveness of publicly funded home-care reablement and highlighted major definitional and evidentiary gaps. In stroke rehabilitation, Qin et al. found a short-term benefit in basic ADLs when home-based rehabilitation was added to usual care, whereas other comparisons were not clearly superior, and the evidence quality was low [17]. Chen et al. reported benefits of reablement for ADL and IADL but no significant effects on quality of life, hospitalization, or unplanned emergency-department use [18]. Collectively, these studies establish clinical and service relevance but also illustrate why policy- and system-level conditions should not be inferred from intervention-effectiveness evidence alone [13,14,15,16,17,18].

Despite this growing policy relevance, much of the occupational therapy literature has focused on clinical effectiveness, profession-specific practice, or local service implementation [11,13,14,15,16,17,18]. Such evidence is important, but it does not, by itself, establish how occupational therapy is institutionally recognized, financed or reimbursed, referred to and prioritized, embedded within multidisciplinary pathways, or supported through workforce and implementation infrastructure [11,12,16]. These organizational and policy conditions operate beyond the individual clinical encounter and can shape whether services are available, accessible, coordinated, and sustainable in practice [2,3,9]. The WHO integrated-care framework explicitly treats governance and coordinated service-delivery arrangements as system requirements for people-centered care [2]. The later WHO report reinforces the need to strengthen integrated service organization across health systems [3]. WHO rehabilitation guidance similarly identifies governance, financing, workforce, and service delivery as health-system actions required to strengthen rehabilitation [9]. A systematic review focused specifically on policy- and system-level evidence is therefore warranted to identify enabling and constraining arrangements, compare them across jurisdictions, and distinguish documented organizational structures from downstream effects that remain uncertain [2,3,9,12].

Accordingly, this review did not conduct a statute-by-statute legal analysis or a comprehensive mapping of government policy documents. Instead, it systematically synthesized peer-reviewed empirical, analytical, and structured implementation evidence that directly addressed occupational therapy at the policy or service-system level. Eligible evidence covered institutional recognition and role definition; financing, coverage, and reimbursement; referral, prioritization, and coordination; service-delivery organization and infrastructure; workforce capacity and implementation support; and policy-relevant access, equity, and user-participation issues. Evidence limited to individual clinical effectiveness without a substantive policy or system component fell outside the review scope. This boundary was applied consistently across the search, eligibility assessment, data extraction, framework synthesis, and interpretation of findings.

Recent institutional reform in the Republic of Korea on integrated community care underscores the need to clarify professional roles, referral pathways, and interagency coordination structures as linkages among health care, long-term care, and community services are strengthened [19]. In this context, occupational therapy should be considered part of the functional and institutional design of integrated community care systems rather than a stand-alone service [2,9,12,19].

This study aimed to systematically characterize and synthesize the policy and system conditions that shape the positioning and implementation of occupational therapy within community-based integrated care, and to derive an evidence-informed analytic framework to support cross-jurisdictional comparison. The review was designed not to determine whether occupational therapy is clinically effective in general, but to answer the prior system question of how function-centered occupational therapy services become recognized, financed, accessed, coordinated, staffed, and sustained within real-world community care arrangements. To this end, five research questions (RQ1–RQ5) were addressed. First, how is occupational therapy described in policy and institutional terms within community-based integrated care systems? Second, what roles and functions are attributed to occupational therapy at the policy and service-delivery levels? Third, how do financing, coverage, referral pathways, and service-delivery systems for occupational therapy vary across countries and institutional contexts? Fourth, how is the integration of occupational therapy connected to continuity of care, sustained community living, function and participation, prevention, and long-term care demand management? Fifth, what are the key conditions supporting the positioning and implementation of occupational therapy within community-based integrated care systems?

Together, these research questions informed a review-based framework for organizing comparisons of the institutional positioning and implementation of occupational therapy across community-based health and social care systems.

## 2. Materials and Methods

### 2.1. Study Design

This study was conducted as a systematic review and reported in accordance with PRISMA 2020 [20]. A systematic review design was selected rather than a scoping review because the review addressed explicitly defined research questions, applied explicit eligibility criteria, used duplicate review procedures with inter-reviewer agreement assessed using Cohen’s kappa [21], employed design-matched critical appraisal, and sought to interpret the strengths and limitations of the evidence concerning defined policy and system arrangements rather than merely mapping the extent and characteristics of the literature. Because the included evidence was heterogeneous in terms of study design, jurisdiction, service context, exposure, and outcome definition, meta-analysis was not appropriate. Therefore, a structured narrative synthesis was used to organize and compare the evidence [22].

The Open Science Framework record (https://doi.org/10.17605/OSF.IO/P7WYD; registered 29 April 2026) was created after the initial bibliographic search and is described as a retrospective methodological record rather than a prospectively registered protocol. The record was subsequently updated to document methodological revisions and the final review procedures. In accordance with PRISMA 2020 Item 24c, changes from the initial approach and the retrospectively created OSF record are explicitly reported. Subsequently, the bibliographic search strategy was redesigned and rerun; the databases searched and the publication period were revised; and eligibility was restricted to peer-reviewed evidence, resulting in the exclusion of non-peer-reviewed grey literature from the final evidence base. The screening procedure was also revised to incorporate a finalized operational eligibility codebook, independent dual screening, and consensus-based reconciliation of title and abstract screening decisions. The synthesis was further strengthened through design-matched critical appraisal, structured framework synthesis, sensitivity analyses, and GRADE-CERQual or GRADE assessments, as applicable. These changes were made to improve disciplinary coverage, reproducibility, the consistency of study selection, and alignment between the research questions and the policy- and system-level evidence base. They were implemented before the revised screening, appraisal, and synthesis reported in this manuscript were finalized. Accordingly, these methods are described as revised or finalized review procedures rather than as prospectively registered protocol elements.

The five research questions and the operational eligibility codebook used in the revised review were finalized as part of these methodological revisions, before the revised screening, final data extraction, framework synthesis, and critical appraisal were conducted. They were therefore specified prospectively in relation to the final screening and synthesis procedures but were not prospectively registered before the original search or the initiation of the review.

### 2.2. Eligibility Criteria for Study Inclusion

For this review, community-based integrated care systems encompassed coordinated primary care, home care, community rehabilitation, social care, home- and community-based services/long-term services and supports (HCBS/LTSS), and hospital-to-community arrangements organized to support continuity outside inpatient- or residential-only settings. Here, LTSS refers to long-term services and supports delivered in home and community settings rather than in institutional long-term residential care. For eligibility, policy- and system-level evidence was operationalized as peer-reviewed evidence concerning at least one OT-related institutional, financing, access, referral/coordination, service-organization, workforce, governance, or implementation arrangement that could influence how occupational therapy was positioned or delivered within a community-based care system. Accordingly, “policy-level evidence” in this review refers to peer-reviewed evidence that describes or analyzes policy-relevant institutional and service-system arrangements, rather than to a systematic review of primary policy documents themselves. Primary government policies, statutes, payer rules, regulatory documents, health-system guidance, and other non-peer-reviewed primary policy materials were outside the final evidence base. A report was included only when all mandatory domains were satisfied: (1) a community-based or hospital-to-community context with explicit community referral, transition, follow-up, or service integration; (2) explicit and separately analyzable OT relevance; (3) at least one substantive OT-related policy- or system-level finding that was separately analyzable and concerned institutional positioning, financing or coverage, referral or coordination, access, service organization, workforce, governance, or implementation; (4) an eligible empirical, analytical, or structured implementation/service-development design; (5) peer-reviewed journal publication; (6) English language; (7) complete full text; and (8) publication from 1 January 2010 to 29 July 2026. Structured implementation/service-development reports were eligible when an implemented OT service or program, identifiable operational data, and traceable OT-related system findings were reported. Reviews, conference abstracts, non-peer-reviewed grey literature, non-analytical professional opinion, and studies limited to individual clinical effectiveness without a policy/system component were excluded. The operational criteria are summarized in Table 1. Multiple reports from one project were retained only when they addressed substantively different questions or outcomes.

### 2.3. Search Strategy

The final databases were PubMed/MEDLINE, CINAHL/EBSCOhost, Scopus, ProQuest Central, and APA PsycINFO. All searches were updated through 29 July 2026 and limited to English-language, peer-reviewed literature published from 1 January 2010 onward. The 2010 start date was selected to focus the review on contemporary policy- and system-level evidence relevant to current community-based and integrated-care arrangements, and this criterion was applied consistently across all databases. The strategy comprised three concept blocks—occupational therapy, community-based integrated-care context, and policy/system-level factors—linked with AND (Table 2). Synonyms and related service or policy terms within each block were linked with OR to maximize sensitivity. Complete database-specific search strings, including field tags and syntax, are provided in Appendix A.

### 2.4. Study Selection

Study selection was conducted independently by the primary reviewer and a second external reviewer, Yun-Hee Park, a doctorally qualified occupational therapist from the Department of Occupational Therapy, Choonhae College of Health Sciences, Republic of Korea, using the operational eligibility codebook finalized before the revised screening process described in Appendix B. She had more than 15 years of professional and research experience in community-based occupational therapy, rehabilitation, and evidence synthesis. She independently conducted title/abstract screening, full-text eligibility assessment, data extraction, framework coding, and design-matched critical appraisal, and participated in consensus discussions to resolve discrepancies between reviewer decisions. She had no supervisory or financial relationship with the author and reported no conflict of interest related to this review. Her contribution was limited to these review procedures and did not extend to study conceptualization, manuscript drafting or substantive intellectual revision, final approval of the submitted manuscript, or acceptance of accountability for the work as an author.

Title/abstract screening was intentionally sensitive during the initial independent screening stage: a record was retained whenever either reviewer considered it potentially eligible or could not confidently exclude it based on the title and abstract alone. After duplicate removal, all 10,271 unique records underwent independent title/abstract screening by both reviewers. At this stage, both reviewers retained 1174 records, Reviewer 1 alone retained 310 records, Reviewer 2 alone retained 236 records, and both reviewers excluded 8551 records. The pre-reconciliation agreement was 94.7%, with a Cohen’s κ of 0.780 [21]. Records independently excluded by both reviewers were not advanced, whereas the 1720 records retained by at least one reviewer underwent a second, consensus-based title/abstract reconciliation.

During reconciliation, both reviewers jointly re-examined the available bibliographic information and title/abstract content using the same operational eligibility criteria that had been finalized before the revised screening process. A record was excluded at this stage only when both reviewers agreed that the available information clearly failed to meet at least one mandatory eligibility criterion; records for which disagreement or uncertainty remained were advanced to full-text assessment. Thus, a unilateral exclusion judgment was insufficient to exclude a record retained by the other reviewer. The reconciliation stage was conducted entirely at the title/abstract level and was distinct from the formal full-text eligibility assessment.

During full-text assessment, both reviewers independently included 19 reports; Reviewer 1 alone included 3 reports, Reviewer 2 alone included 1 report, and both reviewers excluded 15 reports. Pre-consensus agreement at this stage was 89.5%, with a Cohen’s kappa of 0.788 [21]. Disagreements were resolved by jointly re-examining the full texts using the same eligibility codebook, resulting in a final consensus of 21 included and 17 excluded reports. Appendix B provides the operational decision rules applied throughout screening and full-text assessment.

### 2.5. Data Extraction and Preparation for Synthesis

Two reviewers independently extracted data using a standardized workbook. Extracted items included study, country/jurisdiction, care context, study design, sample/data source, OT-related focus, and primary policy/system domain. After extraction, the reviewers compared their entries, re-examined the original reports to resolve discrepancies, and entered consensus values into the final extraction matrix (Appendix C). The extracted data served as the basis for comparing study characteristics and patterns across the included evidence and for the subsequent narrative synthesis [22].

### 2.6. Framework Synthesis

Framework synthesis was conducted using a structured, matrix-based approach. Gale et al.’s Framework Method informed systematic charting and comparison of data across studies and categories [23], while Carroll et al.’s best-fit framework synthesis informed the use of an initial conceptual scaffold that could be refined by the included evidence [24,25]. The initial analytic framework was developed deductively from RQ 1–RQ 5, the policy- and system-related concepts defined in the Introduction, the eligibility criteria and search strategy, and the multilevel, people-centered conceptualization of integrated care [2,5,6]. This process generated eight provisional categories: policy recognition/institutional position; financing/coverage; referral/coordination; service delivery; workforce/implementation; access/equity; governance/collaboration; and system outcomes. These categories served as an initial analytic scaffold for organizing the included evidence and were not treated as fixed final domains.

The included studies were charted against the provisional categories using the extraction matrix and the original reports, and findings were compared across studies and jurisdictions. Overlapping categories were merged, broad categories were split or reframed, and findings spanning multiple areas were retained as cross-cutting considerations. Contradictory and jurisdiction-specific findings were also retained for comparison. Category refinement was based on analytical distinctiveness, recurrence across the included evidence, and the substantive content of directly documented policy- and system-level findings, rather than on frequency alone. Sensitivity analyses were used to assess the robustness of the category structure, and GRADE-CERQual and conventional GRADE assessments were used, where applicable, to calibrate the strength of interpretation of synthesis findings. Comprehensive conceptual saturation was not claimed. Coding and matrix comparisons were managed within the standardized review workbook, and no separate qualitative analysis software was used. The stepwise analytic procedure and category-refinement criteria are summarized in Table 3, and study-level coding, transformations, and decision rationales are provided in Appendix D.

During refinement, governance/collaboration was redistributed across policy recognition/institutional positioning, referral/coordination, and workforce/implementation, based on the substantive content of the contributing findings; accessibility/equity and user participation were retained as a cross-cutting consideration because these issues recurred across multiple domains; and system outcomes were kept separate because they represented measured or hypothesized consequences rather than policy/system arrangements themselves. Accordingly, the final five domains were not obtained by simply reducing the eight provisional categories; they were retained as higher-order organizing domains following study-level charting, category refinement, cross-study and cross-jurisdictional comparison, and robustness assessment.

### 2.7. Critical Appraisal

Critical appraisal was matched to the study design. Qualitative studies were assessed using the JBI Critical Appraisal Checklist for Qualitative Research [26]; analytical cross-sectional studies using the JBI analytical cross-sectional checklist [27]; uncontrolled pre–post and feasibility studies using the JBI quasi-experimental checklist [28]; mixed-methods and quantitative descriptive studies using MMAT 2018 [29]; and Q-methodology, the structured implementation/service-development report, and the legal/policy document analysis using QuADS [30]. For Bolt et al. [31], the descriptive survey and professional-position components were appraised separately using MMAT quantitative descriptive criteria and the JBI expert-opinion approach [32], respectively. Table 4 summarizes the design-matched appraisal approach. JBI judgments were recorded as Yes, No, Unclear, or Not Applicable; MMAT as Yes, No, or Cannot Tell; and QuADS items from 0 to 3. No common cross-design total score or arbitrary quality threshold was used. Item-level judgments are provided in Appendix E.

Two reviewers independently applied the design-matched appraisal criteria to each included report. Disagreements in appraisal were resolved by re-examining the full text and the relevant tool guidance until consensus was reached. Appraisal findings were incorporated into the synthesis at the claim level: descriptive surveys supported reported patterns or perceptions; uncontrolled studies supported feasibility or observed local change but not causal effectiveness; legal/policy analysis supported interpretation of rights and resource allocation but not service effects; professional recommendations were separated from empirical findings; and downstream outcomes not directly measured in the source studies were retained only as hypotheses for future testing.

### 2.8. Assessment of Confidence in Qualitative Findings and Certainty of Quantitative Outcomes

Confidence in individual findings from the qualitative evidence synthesis was assessed using GRADE-CERQual [33,34]. For each synthesis finding, methodological limitations, coherence, data adequacy, and relevance were examined, and overall confidence was rated as high, moderate, low, or very low in accordance with the relevant CERQual guidance [35,36,37,38]. These assessments were used to inform the interpretation of the synthesis findings rather than as a binary criterion for determining whether an included study contributed policy- or system-level evidence. GRADE-CERQual was not applied to quantitative survey findings, directly documented policy or institutional arrangements, expert-opinion statements, or narrative implementation descriptions. Component-level judgments and overall confidence ratings are presented in Appendix G.

Conventional GRADE was applied only to bodies of evidence for quantitatively defined outcomes involving effects, associations, or post-implementation changes [39,40]. Risk of bias, inconsistency, indirectness, imprecision, and publication bias were considered when determining the certainty of evidence for each outcome. GRADE certainty ratings were not assigned to policy or system arrangements, to workforce distributions lacking comparable quantitative estimates, or to downstream system outcomes that were not directly measured. The overall GRADE assessment for each quantitative outcome is presented in Appendix H.

### 2.9. Sensitivity Analysis Procedures

Six sensitivity analyses were conducted: (1) excluding expert-opinion and author-proposed recommendations while retaining empirical findings; (2) restricting the synthesis to findings that were directly documented or measured; (3) removing outcomes that were not directly measured; (4) treating companion reports derived from the same underlying dataset as a single dataset; (5) restricting structured implementation/service-development reports to traceable operational evidence from implemented services, including electronic records, scheduling, billing, and workflow; and (6) restricting uncontrolled implementation studies to feasibility and observed local change rather than causal effectiveness.

## 3. Results

### 3.1. Literature Search and Selection

A total of 15,402 records were identified from five databases: PubMed/MEDLINE (*n* = 5719), CINAHL/EBSCO (*n* = 3634), Scopus (*n* = 4038), APA PsycINFO (*n* = 1141), and ProQuest Central (*n* = 870). After 5131 duplicate records were removed, 10,271 unique records underwent title/abstract screening. During the initial title/abstract screening stage, both reviewers retained 1174 records, Reviewer 1 alone retained 310 records, Reviewer 2 alone retained 236 records, and both reviewers excluded 8551 records. Pre-reconciliation agreement was 94.7%, with a Cohen’s κ of 0.780 [21]. The 1720 records retained by at least one reviewer proceeded to title/abstract reconciliation. Following reconciliation, an additional 1682 records were excluded, leaving 38 reports for formal full-text eligibility assessment. Thus, 10,233 records were excluded before full-text assessment.

**Figure 1:**
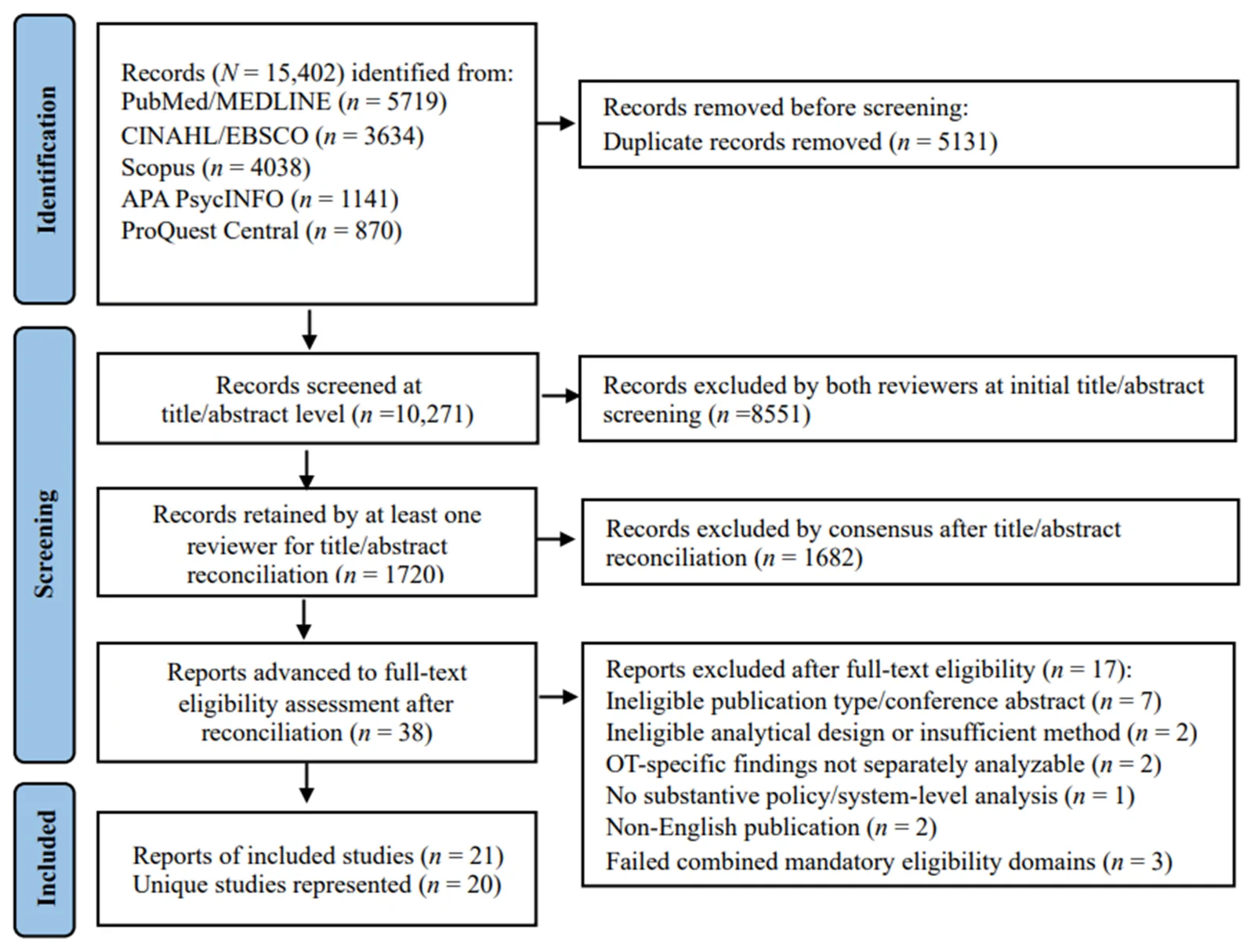
PRISMA flow diagram of the study identification, screening, eligibility, and inclusion process.

At the full-text eligibility stage, 17 reports were excluded. Reasons for exclusion included ineligible publication type or conference abstract (*n* = 7), ineligible analytical design or insufficient methodological detail (*n* = 2), OT-related findings not separately analyzable (*n* = 2), no substantive policy- or system-level analysis (*n* = 1), non-English publication (*n* = 2), and failure to meet multiple mandatory eligibility domains (*n* = 3). Twenty-one reports were ultimately included in the review. Two companion publications derived from the same underlying program dataset were retained because they addressed distinct research questions and reported different findings; however, to avoid double counting, they were treated as a single study at the unique study/dataset level. Accordingly, the 21 included reports represented 20 unique studies/datasets (Figure 1).

Before consensus at the full-text stage, both reviewers independently included 19 reports: Reviewer 1 alone included 3 reports, Reviewer 2 alone included 1 report, and both reviewers excluded 15 reports. Pre-consensus agreement was 89.5%, with a Cohen’s κ of 0.788. Disagreements were resolved by re-examining the full texts using the same operational eligibility criteria and coding rules, resulting in a final consensus on 21 included and 17 excluded reports.

### 3.2. Characteristics of Included Evidence

The 21 reports were published between 2010 and 2025. Jurisdictions included the United States (*n* = 6), Canada (*n* = 5), Australia (*n* = 4), the United Kingdom (*n* = 3), South Korea (*n* = 1), Europe/COTEC (*n* = 1), and a global WFOT survey (*n* = 1). Study designs included qualitative case or interview studies, quantitative descriptive and cross-sectional surveys, mixed-methods and Q-methodology studies, uncontrolled implementation evaluations, policy-document analysis, a legal/ethical analysis, and a peer-reviewed report that combined an empirical survey of member organizations with a professional-position component.

Settings covered primary care, reablement, home care, community health, Medicaid HCBS, LTSS care coordination, adult social care, integrated health and social care, and home-based rehabilitation. Table 5 summarizes the country/jurisdiction, care context, design/data source, OT-related focus, and principal policy/system contribution for all 21 reports; more granular extraction fields are provided in Appendix C. For Bolt et al. [31], the empirical survey component included responses from 20 of the 30 COTEC member associations. Only the separately analyzable findings from the member-association survey were treated as empirical evidence, whereas the normative recommendations reflecting professional positions were considered separately.

### 3.3. Critical Appraisal and Interpretive Restrictions

Critical appraisal identified recurrent design-specific limitations rather than a single quality hierarchy. Among the five qualitative studies, the research question, data collection, analysis, ethics, and conclusions domains were generally well supported, whereas researcher positioning, influence, reflexivity, and transferability were incompletely reported in several studies. The four analytical cross-sectional studies provided useful data on service patterns and associations, but selection, representativeness, measurement, confounding, and transferability were frequently unclear. The two quasi-experimental or feasibility studies lacked concurrent controls; Missen et al. also had concurrent staffing changes, and Dickson and Toto used a small, selected sample. Mixed-methods studies commonly used non-probability sampling and incompletely reported integration or discordant findings. Quantitative descriptive surveys were limited by uncertain representativeness, non-response, and measurement validation. QuADS appraisal identified comparatively complete reporting for Lim et al. (35/39), intermediate reporting for Carrier et al. (28/39), and limited methodological transparency for Koverman et al. (22/39); these totals were descriptive only and were not converted into quality grades.

Critical appraisal was used to define how each report could contribute to the synthesis rather than to rank the evidence with a single overall quality score. Table 6 therefore retains the report-level appraisal information needed to understand the results: the design-matched appraisal tool, the principal methodological limitation, the evidentiary contribution, and the restriction applied during interpretation. Full final item-level judgments are provided in Appendix E. Koverman et al. was used only for traceable operational arrangements and implementation experience; Carrier et al. for legal/policy interpretation rather than empirical outcome effects; and Bolt et al.’s survey findings were interpreted separately from their professional position statements.

### 3.4. Cross-Jurisdictional Patterns

Cross-jurisdictional comparison showed that OT integration was not organized around a single transferable model. U.S. evidence focused on reimbursement, billing, Medicaid HCBS design, and entry into primary-care or LTSS programs; Canadian evidence differentiated between Ontario Family Health Team integration and Quebec home-care prioritization, waiting-list management, and legal/ethical allocation; Australian evidence emphasized community health delivery, rural access, outreach, and workforce constraints; U.K. evidence emphasized reablement, integrated health-social care roles, and personalization; the Korean study focused on role perceptions in home-based rehabilitation; and the global/WFOT and European/COTEC surveys demonstrated wide variation in financing, service settings, referral routes, and workforce capacity.

**Figure 2:**
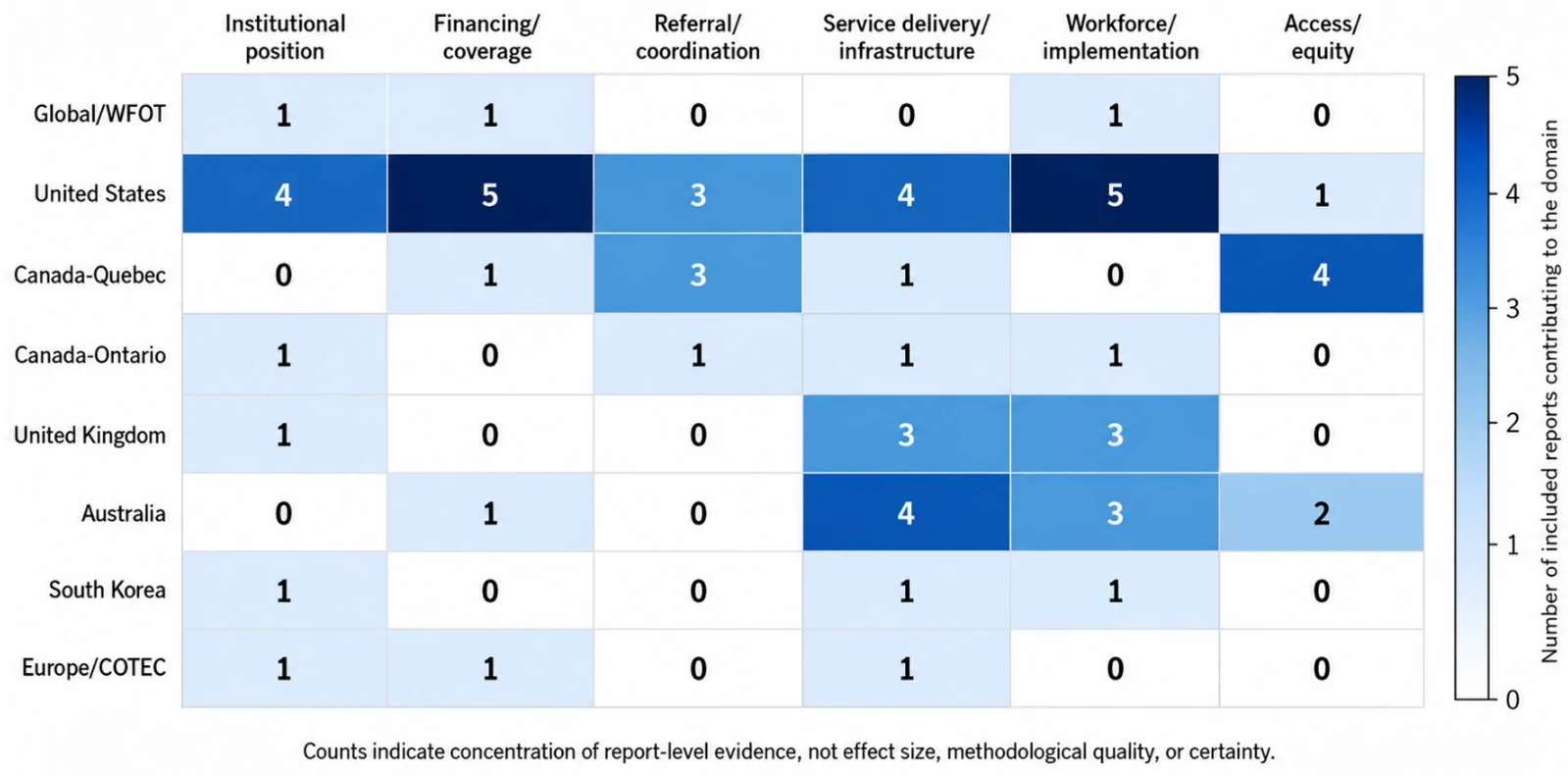
Distribution of included reports across the final policy-system domains and the cross-cutting accessibility/equity consideration, by jurisdiction. Cell values were derived from the final study-level domain mappings presented in Appendix D. Each report was counted once in each domain to which it contributed; therefore, reports could contribute to multiple domains. The counts indicate the concentration of evidence rather than effect magnitude, methodological quality, or certainty of evidence. COTEC, Council of Occupational Therapists for the European Countries; WFOT, World Federation of Occupational Therapists.

Table 7 compares jurisdictional groupings across care contexts, distinctive policy/system patterns, major barriers or enablers, and interpretive boundaries. Figure 2 summarizes the concentration of report-level evidence using the final study-level domain mappings documented in Appendix D. Each report was counted once in each final domain to which it contributed; therefore, a single report could contribute to more than one domain. The accessibility/equity column represents the corresponding cross-cutting consideration recorded in Appendix D. The counts indicate the concentration of evidence rather than effect magnitudes, methodological quality, or certainty of evidence.

Contrasting findings were retained rather than resolved into a single pattern. Formal or professional recognition of OT did not consistently translate into service availability, and organizational integration supports, such as co-location or shared records, were not uniformly present across jurisdictions.

### 3.5. Findings by Research Question

Across the five review questions, OT was institutionally positioned through heterogeneous arrangements across primary care, home and community care, HCBS/LTSS, reablement, social care, and home rehabilitation. OT-related functions centered on contextualized functional and ADL/IADL assessment, environmental modification, participation-oriented intervention, reablement, self-management, caregiver support, assistive technology, coordination, and team education. Financing, coverage, referral, prioritization, waiting-list management, shared records, staffing, and local service organization varied substantially across jurisdictions. Directly measured system outcomes were limited mainly to waiting time, access/attendance, service activity, projected expenditure/utilization, workforce time use, and implementation feasibility; hospitalization, institutionalization, cost savings, and long-term care demand were not established as effects of OT integration. Across these questions, five recurrent policy-system domains were identified, with access, equity, and user participation operating across domains (Table 8).

### 3.6. Sensitivity Analyses

Sensitivity analyses did not alter the five-domain structure. Excluding professional or normative recommendations, all five domains remained identifiable. Restricting the synthesis to directly documented findings removed or weakened broader claims about hospitalization, institutionalization, costs, and long-term care demand, while preserving claims about financing, referral, access, service delivery, and workforce patterns. Treating companion publications as a single dataset reduced the apparent volume of referral/access evidence without changing the synthesis. Restricting structured implementation reports and uncontrolled studies to traceable operations, feasibility, or observed local change removed unsupported causal interpretations.

The main consequence of the sensitivity analyses was therefore interpretive rather than structural: claims were limited to the level directly supported by the contributing study designs, while downstream system outcomes remained priorities for future empirical testing. Table 9 summarizes these robustness checks and their implications; detailed decision rules and changes in interpretation are provided in Appendix F.

### 3.7. Confidence in Qualitative Findings and Certainty of Quantitative Outcomes

GRADE-CERQual was applied to six synthesis findings supported by qualitative studies or by separately analyzable qualitative components. Based on an overall assessment of methodological limitations, coherence, adequacy of data, and relevance, three findings—role clarity and team understanding; co-location, shared records, team meetings, and managerial support; and the influence of service context and workforce capacity on OT roles—were rated as having moderate confidence. Three findings—reimbursement and billing constraints; differences in referral prioritization and fairness judgments among stakeholders; and tensions between service redesign and occupation-centered practice—were rated as having low confidence.

Conventional GRADE was applied to three quantitative outcomes: changes in waiting time, the association between waiting-list management and waiting status, and the feasibility of integrating OT into care coordination. Given the risk of bias, indirectness, imprecision, and related limitations, all three outcomes were rated as having very low certainty of evidence. Accordingly, observed changes or associations were reported but not interpreted as establishing causal effectiveness. GRADE ratings were not assigned to directly documented policy- or system-level arrangements. The key confidence and certainty judgments and their interpretation are summarized in Table 10, while the detailed CERQual and GRADE evidence profiles are presented in Appendix G and Appendix H, respectively.

### 3.8. Integrated Policy-System Synthesis Refined Through Framework Synthesis

The final policy-system domains were refined through framework synthesis by charting data from the 21 included reports against the eight provisional deductive categories and iteratively comparing and reorganizing the categories across studies and jurisdictions. Sensitivity analyses were used to assess the robustness of the category structure after limiting the influence of professional recommendations, unmeasured outcomes, companion publications, and uncontrolled studies. GRADE-CERQual and conventional GRADE assessments were used to calibrate the strength of the interpretation of the synthesis findings.

**Figure 3:**
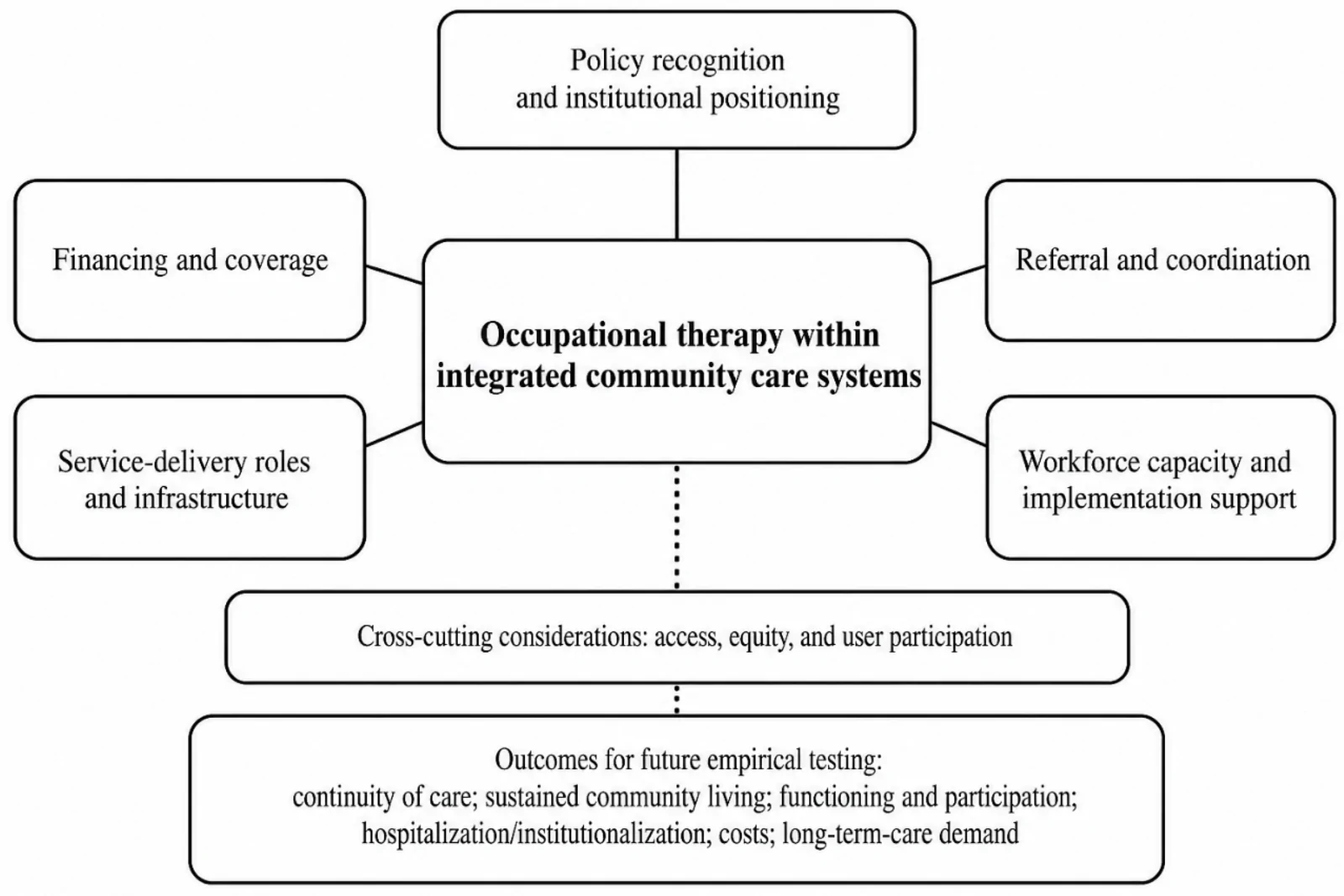
Evidence-informed organizing framework for the policy- and system-level positioning of occupational therapy within integrated community care systems.

This process yielded five higher-order policy-system domains: (1) policy recognition and institutional positioning, (2) financing and coverage, (3) referral and coordination, (4) service-delivery roles and infrastructure, and (5) workforce capacity and implementation support. Accessibility, equity, and user participation were classified as cross-cutting considerations because they spanned multiple domains rather than forming a separate domain. Table 11 summarizes the recurring evidence patterns, OT-related system contributions, and remaining uncertainties within each domain. Figure 3 presents the resulting organizing framework, illustrating the interrelated nature of the five domains and the cross-cutting role of accessibility, equity, and user participation. The connections between domains do not imply a fixed sequence or causal pathway, and the links to downstream system outcomes are hypotheses that require future empirical evaluation. Study-level transformations from the provisional categories to the final domains, along with the corresponding decision rationales, are provided in Appendix D.

The five policy-system domains are higher-order domains refined through framework synthesis of the included evidence. Accessibility, equity, and user participation serve as cross-cutting considerations across the domains. Solid connections indicate conceptual relationships among the domains rather than causal pathways, whereas dotted connections to downstream system outcomes represent hypotheses that require future empirical testing.

## 4. Discussion

### 4.1. Principal Findings and the Contribution of Occupational Therapy

The central finding of this review is that integrating occupational therapy (OT) into community-based care is better understood as an alignment problem across policy and system arrangements than as the simple addition of one profession to a team. Across the 21 included reports, institutional recognition, financing and coverage, referral and coordination, service-delivery infrastructure, and workforce and implementation support recurred as interrelated dimensions. However, the evidence did not establish that all five are universally necessary or sufficient, operate in a fixed sequence, or carry equal importance across jurisdictions. This interpretation is consistent with broader integrated-care evidence showing substantial heterogeneity in effects and costs across models and contexts [7,8,60,61] and with rehabilitation reviews emphasizing that service integration remains context-dependent [62,63]. It also aligns with WHO’s health-system approach to rehabilitation, which emphasizes governance, financing, service delivery, workforce, and information as system components requiring coordinated strengthening [9]. Recent health policy and systems research (HPSR) scholarship similarly argues that rehabilitation integration requires attention to how policy, financing, governance, implementation, and service organization operate in real health systems [64]. Thus, the contribution of this review is to provide an OT-relevant organizing structure that distinguishes directly documented policy-system arrangements from downstream outcomes that remain uncertain.

Within these broader system mechanisms, OT’s contribution was most evident in translating functional and occupational information into community-care pathways. The included evidence repeatedly described contextualized functional and ADL/IADL assessment, home and environmental modification, reablement, participation, self-management, assistive technology, caregiver support, and coordination across teams and services [12,31,42,43,44,45,49,52,54,55,57,59]. This pattern aligns with primary-care OT literature on prevention, chronic-condition management, functional assessment, environmental modification, self-management, and collaborative service delivery [11,65]. The contribution of OT in the included evidence therefore appears not to lie in creating a separate system mechanism, but in embedding function-, occupation-, participation-, and environment-focused content within financing, referral, organizational, and workforce arrangements. Evidence-certainty assessments constrain these interpretations. The qualitative synthesis findings showed moderate or low CERQual confidence, indicating that recurrent patterns could be identified but should be generalized cautiously across settings. All quantitative outcomes were rated as having very low GRADE certainty, precluding interpretation of the observed changes or feasibility findings as causal effects. Accordingly, the synthesis emphasizes the documented presence and recurrent patterns of policy- and system-level arrangements, with interpretation calibrated to the certainty and design of the contributing evidence.

### 4.2. Financing, Access, and Equity

Financing and access findings revealed a persistent gap between formal recognition and practical availability. Across the included reports, funding sources, reimbursement, waiver design, billing procedures, and local resource constraints shaped whether OT could be offered and sustained [12,41,45,50,51,52,59]. Access was filtered through referral priorities, waiting-list rules, geography, outreach, transportation, and health literacy [46,47,48,53,56,58]. These mechanisms are interconnected: financing influences service capacity, while referral and prioritization arrangements determine how scarce capacity is distributed among potential users.

The broader evidence supports a cautious development agenda rather than a single preferred financing model. A 2026 Cochrane overview of financial arrangements for rehabilitation found extremely sparse evidence and could not identify a reliable financing approach that policymakers should preferentially adopt [66]. Reviews of rural primary care and rehabilitation access likewise identify workforce, financing, transportation, technology, policy, and service-delivery constraints as interacting determinants of access [65,67,68,69]. For OT service development, the defensible implication is to make eligibility, covered functions, reimbursement or payment routes, and referral responsibility explicit, then evaluate whether the chosen arrangements improve reach, waiting time, continuity, and financial and distributive equity. The stakeholder differences identified in the included OT evidence [48,56,58] further indicate that equity should be evaluated in terms of who receives timely access and whose priorities are reflected in allocation decisions, rather than assumed to follow automatically from service integration.

### 4.3. Organizational Integration and Workforce

OT studies consistently link organizational integration to shared records, co-location or outreach, team meetings, communication, role clarity, managerial support, peer consultation, and protected time for indirect coordination work [42,43,45,54,55]. Broader interprofessional literature identifies comparable mechanisms at the organizational, team, and individual levels [70,71,72,73], and transitional-care evidence likewise highlights communication, coordination, professional capacity, and implementation context [74]. This convergence supports interpreting OT integration as organizational work involving role design and interprofessional processes, rather than merely adding funded posts.

Recent rehabilitation HPSR underscores the need for caution and evaluation. A 2026 Cochrane overview found limited, predominantly low-certainty evidence on delivery arrangements, with most evidence from high-income countries [75]. A companion overview of implementation strategies likewise found generally low-certainty evidence and sparse reporting of organizational, economic, implementation, and equity outcomes [76]. Accordingly, service expansion should preferably proceed through staged, evaluable implementation, with referral workflows, information exchange, role clarity, leadership, training, coordination time, and workforce capacity specified in advance and monitored during scale-up. The current OT evidence does not support transferable staffing thresholds or a single optimal organizational configuration.

### 4.4. Potential Policy and Service-Planning Considerations

Taken together, the reviewed OT evidence and external rehabilitation-system evidence support a staged, evaluable approach to policy and service development rather than a universal implementation prescription. At a minimum, service planners can make the institutional mandate and OT eligibility explicit and align coverage and payment with defined community-based OT functions. Referral, triage, information-sharing, and transition pathways should also be specified, along with workforce, leadership, training, and coordination support. These elements closely parallel WHO priorities for integrating rehabilitation into health systems [9] and the HPSR agenda for understanding how rehabilitation is embedded within governance, financing, delivery, and implementation arrangements [64]. However, a 2025 Cochrane overview found no eligible systematic-review evidence to estimate the effects of rehabilitation governance arrangements [77]. Therefore, institutional mandates, accountability mechanisms, and coordination rules should be treated as context-specific design choices to be monitored and evaluated, not as proven mechanisms of effectiveness.

Equity and participation should be built into this development process rather than assessed only after implementation. Service-user and caregiver co-design literature suggests that participation can improve alignment between service models and complex needs, though the depth and quality of participation vary [78,79]. This is particularly relevant where OT supply, transport, geography, socioeconomic disadvantage, cultural relevance, or health literacy may constrain access. Before large-scale expansion, jurisdictions should define a core evaluation set that includes referral completion, waiting time, equitable reach, function and ADL/IADL, participation, caregiver outcomes, continuity, service utilization, and costs. A recent scoping review found that outcome evaluation in primary-care OT remains multifaceted and inconsistent, with cost-related evaluation particularly underrepresented [80]. Standardized routine measurement would therefore strengthen both local quality improvement and cross-jurisdictional learning. Hospitalization, institutionalization, and long-term care demand should remain prospective outcomes of testing rather than assumed benefits of OT integration.

### 4.5. Strengths and Limitations

This review has several strengths: an expanded multidisciplinary database search updated through July 2026; explicit OT-related eligibility criteria; independent selection, data extraction, and design-matched critical appraisal by two reviewers; an operational eligibility codebook finalized before the revised screening process; control of companion publications; cross-jurisdictional and research-question-level synthesis; sensitivity analyses; and separate use of GRADE-CERQual and GRADE for appropriate evidence types. Study-level transformation from provisional categories to final domains was documented, and appraisal findings were carried forward as explicit restrictions on the claims made in the synthesis.

Several limitations remain. First, although the initial title and abstract screening was intentionally sensitive, this sensitivity was achieved by ensuring that no record retained by either reviewer was excluded unilaterally; instead, such records proceeded to consensus-based reconciliation. Of the 1720 records entering reconciliation, 1682 were subsequently excluded without full-text retrieval only after both reviewers agreed that the available bibliographic information and title and abstract clearly failed to meet at least one mandatory eligibility criterion. Records that remained uncertain or for which the reviewers disagreed were advanced to full-text assessment. Nevertheless, because these decisions were made without examination of the complete reports, potentially relevant studies may have been missed when policy- or system-level content was not clearly reflected in the title or abstract.

Second, the OSF record was created retrospectively, after the initial bibliographic search, rather than prospectively registered before the review began. Substantial methodological revisions were subsequently introduced before the final screening, appraisal, and synthesis were conducted. These included revisions to the search strategy and eligibility procedures, as well as strengthening of the critical appraisal and synthesis approaches. Although these changes were documented transparently and applied consistently to the final review procedures, the absence of a prospectively registered protocol means that some methodological decisions may have been informed by knowledge gained as the review evolved.

Third, only English-language, peer-reviewed literature was included, while primary government policies, statutes, payer rules, regulatory documents, health-system guidance, and other grey literature were excluded from the final evidence base. This limitation is particularly relevant to a policy- and system-focused review because formal institutional arrangements may be documented primarily in legislation, government policies, commissioning documents, payer guidance, or health-system reports rather than in peer-reviewed journals. Their exclusion may therefore have resulted in the incomplete capture of formal policy arrangements, particularly recent or jurisdiction-specific changes. Accordingly, the findings should be interpreted as a synthesis of peer-reviewed evidence on policy- and system-level arrangements rather than as a comprehensive mapping of the policy environment.

The evidence base was also concentrated in high-income countries, and many studies relied on convenience samples, professional networks, or small local samples. The search was restricted to a predefined set of major health, rehabilitation, and multidisciplinary bibliographic databases; therefore, relevant studies indexed only in other bibliographic databases or specialized social-policy and social-care sources may have been missed. Some studies used self-developed measures, while uncontrolled implementation studies lacked concurrent comparators. Moreover, perspectives from service users and caregivers, as well as evidence from rural or remote settings, culturally diverse populations, and lower-resource settings, were limited. The framework should therefore be understood as an organizing structure for the available evidence, and its applicability should be considered in light of differences in financing, regulation, workforce, and social-care systems across jurisdictions. Finally, the external WHO guidance and post-search HPSR literature cited in the Discussion were used only to contextualize the review findings and develop implications; they were neither incorporated into nor counted as part of the systematic review evidence base.

### 4.6. Future Research

Future research should move beyond role descriptions to test specific policy and system mechanisms. Comparative natural experiments, prospective controlled studies, interrupted time-series analyses, linked administrative and claims data, and mixed-methods or hybrid implementation-effectiveness designs could evaluate whether changes in payment arrangements, eligibility, referral pathways, shared information systems, workforce configuration, or coordination processes affect equitable access, timeliness, continuity, function and participation, caregiver outcomes, service use, costs, and institutionalization. The major gaps identified by recent rehabilitation HPSR overviews in financing, delivery, governance, and implementation reinforce the need for studies that measure organizational, economic, and equity outcomes rather than clinical outcomes alone. Cross-jurisdictional studies should explicitly model financing, regulation, referral authority, social-care organization, and workforce supply, and should include lower-resource and non-English-language settings. Finally, because outcome measurement in primary-care OT is currently inconsistent, developing a core system- and service-level outcome set would improve comparability and make future scale-up decisions more evidence-informed.

## 5. Conclusions

This systematic review addressed five research questions that structured the final review across 21 reports representing 20 unique studies/datasets. For RQ1, OT was institutionally positioned in primary care, HCBS/LTSS, reablement, community health, home care, care coordination, and home-based rehabilitation, but formal recognition varied across jurisdictions and did not ensure access. For RQ2, recurrent OT functions centered on contextualized assessment of function and participation, ADL/IADL, environmental modification, reablement, self-management, assistive technology, coordination, and support for teams and caregivers. For RQ3, financing, coverage, reimbursement, referral, prioritization, access, service organization, and workforce arrangements varied substantially across jurisdictions. For RQ4, directly documented outcomes focused on waiting time, access or attendance, service activity, projected utilization or expenditure, workforce processes, and implementation feasibility; effects on hospitalization, institutionalization, costs, and long-term care demand were not established. For RQ5, five higher-order domains repeatedly organized the evidence: policy recognition and institutional positioning, financing and coverage, referral and coordination, service-delivery roles and infrastructure, and workforce capacity and implementation support, with accessibility, equity, and user participation operating across domains.

The principal contribution of this review is therefore not confirmation of the effectiveness of a single integration model, but a structured identification of the policy-system arrangements and implementation conditions that shape how OT can be positioned and delivered in community-based care. For policy and service development, the evidence supports considering an evaluable package of explicit institutional positioning and eligibility, financing and coverage, referral and information pathways, workforce and implementation support, and equity- and user-responsive service design. These components should be configured to local governance and resources and tested prospectively rather than treated as a universal checklist. Recent rehabilitation HPSR evidence similarly shows that the effects of financial, delivery, governance, and implementation arrangements remain limited or uncertain, and that OT-related outcome measurement requires greater standardization. The resulting framework should therefore be used as an evidence-informed organizing structure for comparative planning and hypothesis testing rather than as a validated causal model; downstream system outcomes remain priorities for prospective evaluation.

## Figures and Tables

**Table 1:** Eligibility Criteria.

Domain	Inclusion Criteria	Exclusion Criteria
Population/context	Community-dwelling populations; primary care, community rehabilitation, social care, home care, home health, HCBS, reablement, community LTSS; hospital-to-community transitions when separately analyzable.	Hospital, institution, or residential-only settings without community transition, referral, coordination, follow-up, or separately extractable community findings.
OT relevance	OT services, therapists, or workforce explicitly presented; at least one substantive OT-related finding separately extractable.	OT absent, incidental, listed only among professions, or not separable from generic multidisciplinary rehabilitation.
Policy/system relevance	At least one OT-related result in recognition, governance, legislation, eligibility, financing, reimbursement, coverage, commissioning, referral, coordination, access/waiting, workforce, organization, implementation, or resources.	Policy/system terms only in the background/discussion; clinical technique or patient-level effectiveness without an OT-related system finding.
Design/analysis	Original qualitative, quantitative, mixed, health services, implementation, workforce, organizational, comparative, or analytic case study; transparent policy/legal/document analysis.	Review, editorial, commentary, advocacy, opinion, position paper without an identifiable source base and transparent analysis, or descriptive clinical case.
Publication	Peer-reviewed journal article, 1 Jan 2010–29 Jul 2026, English, complete full text.	Grey literature, conference material, protocol, book/chapter, thesis, non-English, unavailable full text, outside period.
Companion reports	May retain distinct reports from one dataset when questions or outcomes differ; link to that dataset.	Exact duplicates or substantially overlapping reports without additional relevant information.

HCBS, Home and Community-Based Services; LTSS, Long-Term Services and Supports; OT, occupational therapy.

**Table 2:** Three concept blocks used in the database search strategy.

Concept Block	Purpose	Core Search-Term Families (Examples)
Occupational therapy	Explicitly identify the target profession	occupational therapy; occupational therapist *; occupational therapy practitioner *
Community-based integrated-care context	Capture primary, community, home, rehabilitation, and social care contexts outside acute inpatient-only care	integrated care; primary care/primary health care; community care/community health; home care; HCBS; community rehabilitation; home-based rehabilitation; social care; LTSS; reablement; transitional care; aging/ageing in place
Policy/system-level factors	Capture institutional conditions that govern how services are positioned, financed, referred to, coordinated, and implemented	policy/policies; governance; legislation/regulation; financing/funding; reimbursement/payment; coverage/eligibility; referral; care coordination; access/accessibility; waiting list/prioritization; workforce/staffing; service delivery/organization; implementation; integration

* indicates a truncation wildcard used to retrieve terms with variant word endings (e.g., therapist and therapists). HCBS, Home and Community-Based Services; LTSS, Long-Term Services and Supports.

**Table 3:** Stepwise framework-synthesis procedure and criteria for category refinement.

Stage	What Was Done in This Review	Decision Criteria	Output
1. Initial analytic framework	Eight provisional categories were developed deductively from RQ1–RQ5, the policy- and system-related concepts defined for the eligibility criteria and search strategy, and the multilevel and people-centered conceptualization of integrated care.	Provisional categories were used as an initial analytic scaffold and were not treated as fixed final domains.	Eight deductively derived provisional categories
2. Study-level charting and category refinement	Included studies were charted using the extraction matrix and original reports. Overlapping categories were merged, broad categories were split or reframed, and findings spanning multiple areas were retained as cross-cutting considerations.	Recurrent concepts that were not analytically separable were merged, while substantively distinct concepts were retained. Directly documented findings were distinguished from interpretations or hypotheses.	Refined candidate domains and cross-cutting considerations
3. Cross-study and cross-jurisdictional comparison	The refined matrix was compared across studies, jurisdictions, and service contexts to examine recurrent patterns, differences, and context-specific findings.	Both recurrence and substantive policy/system distinctiveness were considered; frequency alone did not determine domain importance.	Higher-order candidate policy/system domains
4. Robustness assessment and final integration	Sensitivity analyses were used to assess robustness, and applicable CERQual/GRADE assessments were used to calibrate the strength of interpretation before final integration.	Directly documented policy/system arrangements were distinguished from causal or effect claims.	Final integrated organizing framework

CERQual, Confidence in the Evidence from Reviews of Qualitative Research; GRADE, Grading of Recommendations Assessment, Development and Evaluation; RQ, research question.

**Table 4:** Design-matched critical-appraisal approach.

Design/Evidence Type	Appraisal Tool
Qualitative studies	JBI Critical Appraisal Checklist for Qualitative Research
Analytical cross-sectional studies	JBI Critical Appraisal Tool for Analytical Cross-Sectional Studies
Quasi-experimental/ feasibility studies	JBI Critical Appraisal Checklist for Quasi-Experimental Studies
Mixed-methods studies	MMAT 2018—Mixed Methods
Quantitative descriptive surveys	MMAT 2018—Quantitative Descriptive
Diverse/non-standard designs	QuADS
Professional-position component	JBI Textual Evidence—Expert Opinion

JBI, Joanna Briggs Institute; MMAT, Mixed Methods Appraisal Tool; QuADS, Quality Assessment for Diverse Studies.

**Table 5:** Characteristics and principal policy/system contributions of the 21 included reports.

Study	Country/ Jurisdiction	Care Context	Design and Sample/Data Source	OT-Related Focus	Key Policy/System Contribution
Freeman et al. (2025) [12]	Global/WFOT	Primary care	Cross-sectional survey; 59/100 WFOT member organizations	Global mapping of OT primary-care services, settings, populations, financing, workforce and enabling factors	Policy/institutional position; financing; workforce
Nash et al. (2025) [41]	United States	Primary care	Mixed methods; 41 PCPs, 305 OTs; interviews with 6 PCPs and 1 OT	Receptiveness to OT inclusion, delegation preferences, reimbursement, and role barriers	Institutional position; financing; workforce
Evans et al. (2023) [42]	Australia	Community health	Cross-sectional mixed-methods survey; 80 OTs	Intra-disciplinary OT practice, peer consultation, management support, funding, and organizational barriers	Service delivery; workforce
Dibsdall (2022) [43]	United Kingdom	Reablement/social care	Realist qualitative multiple-case study; 3 reablement services	OT assessment, goal setting, intervention selection, support-worker training, co-location and communication	Service delivery; implementation
Lim et al. (2022) [44]	South Korea	Home-based rehabilitation	Q methodology; 34 rehabilitation-team members	Five role-perception types emphasizing home adaptation, participation, service reliability, client needs, and professional development	Institutional position; service delivery; workforce
Rouch et al. (2022) [45]	United States	Primary care	Descriptive qualitative study; 7 OTs	Entry to primary-care teams, team navigation, patient-centered practice, reimbursement and expansion barriers	Institutional position; financing; referral; workforce
Missen et al. (2021) [46]	Australia	Rural community health	Pre/post service evaluation; 456 clients	Basic Assessment Model using OT/AHA workflow; median wait decreased from 105 to 80 days	Access/waiting; service delivery; workforce
Smith & Gallego (2021) [47]	Australia	Community child health	Mixed methods: routine service data plus parent/carer survey (*n* = 21)	Outreach, attendance, and transport/health-literacy barriers in a disadvantaged area	Access/equity; service delivery
Raymond et al. (2020) [48]	Canada/Quebec	Home-care OT	Qualitative component: 11 OTs, 10 older persons, 9 adults with disabilities	Stakeholder differences in referral-prioritization rationales: safety, independence and social participation	Referral/prioritisation; equity; participation
Bolt et al. (2019) [31]	Europe/COTEC	Primary/community care	Survey of 20/30 COTEC member associations embedded in a position paper	Cross-country service/funding organization plus professional recommendations for primary-care positioning	Institutional position; financing; service delivery
Dickson & Toto (2018) [49]	United States	LTSS care coordination	Single-group feasibility study; 23 referred, 16 evaluated	Feasibility of embedding OT evaluation and goal-based intervention in an aging-in-place care-coordination program	Service delivery; coordination; implementation
Friedman & VanPuymbrouck (2018) [50]	United States	Medicaid HCBS/ LTSS	Mixed quantitative/document analysis; 111 waivers from 46 states and DC	OT waiver coverage, projected utilization, expenditures, reimbursement, and service definitions	Financing/coverage; policy design
Dahl-Popolizio et al. (2017) [51]	United States	Primary care	Descriptive survey with open-ended responses; OTs and PCPs	Receptiveness to OT on primary-care teams and perceived funding/role barriers	Institutional position; service delivery; financing
Koverman et al. (2017) [52]	United States	Geriatric primary care	Structured implementation/service-development report; 8-week pilot	Operational development of OT service using logic model, EMR, scheduling, referral, and reimbursement processes	Service infrastructure; financing; implementation
Raymond et al. (2016) [53]	Canada/Quebec	Home-care OT	Cross-sectional program survey; 55 programs	Waiting-list management strategies for low-priority clients and association with wait-list length	Access/waiting; service management
Wilberforce et al. (2016) [54]	United Kingdom	Integrated health and social care	Cross-sectional time-use pilot; 151 practitioners	Distribution of OT and assistant time across direct care, indirect casework, team/service activity, and therapeutic work	Workforce; service organization
Donnelly et al. (2013) [55]	Canada/Ontario	Primary care	Multiple case studies; 4 Family Health Teams	Organizational structures supporting OT integration: communication, trust, role understanding, co-location, EMR, and team meetings	Institutional position; referral/coordination; service delivery/infrastructure; workforce/implementation
Raymond et al. (2013) [56]	Canada/Quebec	Home-care OT	Survey/content analysis; 55 programs	Referral-prioritization criteria and wide differences in wait times by priority level	Referral/prioritisation; access/equity
London-Willis et al. (2012) [57]	United Kingdom	Adult social care	Qualitative interviews; 6 OTs	Perceived effects of personalization on OT roles, identity, opportunities and threats	Institutional position; workforce; service delivery
Carrier et al. (2010) [58]	Canada/Quebec	Community OT services	Legal, ethical and clinical document analysis	Access, prioritization, waiting, resource allocation, distributive justice, and service quality	Financing/coverage; referral/coordination; accessibility/equity (cross-cutting)
Quick et al. (2010) [59]	Australia/Victoria	Community health	Descriptive postal survey; 101 returned, 72 eligible	Scope of practice, health promotion, funding/resource barriers, and workforce characteristics	Service delivery; workforce; financing

AHA, Allied Health Assistant; COTEC, Council of Occupational Therapists for the European Countries; DC, District of Columbia; EMR, electronic medical record; HCBS, Home and Community-Based Services; LTSS, Long-Term Services and Supports; OT, occupational therapy; OTs, occupational therapists; PCPs, primary care providers; WFOT, World Federation of Occupational Therapists.

**Table 6:** Design-matched critical appraisal and interpretive restrictions used in the synthesis.

Study	Appraisal Tool	Principal Methodological Limitation	Evidence Contribution	Restriction Applied in Synthesis
Freeman et al. (2025) [12]	JBI analytical cross-sectional	Member-organization self-report; representativeness and measurement partly unclear	Cross-jurisdictional service, financing, referral, and workforce patterns	No causal or fully representative global inference
Nash et al. (2025) [41]	MMAT mixed methods	Convenience/snowball sampling; very small interview sample; limited integration	Reported receptiveness and reimbursement/role barriers	Perceptions only; no prevalence or effectiveness claim
Evans et al. (2023) [42]	MMAT mixed methods	Convenience sample; self-developed survey; geographic overrepresentation	Organizational and interdisciplinary practice experiences	Limited national generalization
Dibsdall (2022) [43]	JBI qualitative	Single researcher; limited direct service-user voice and reflexivity	Contextual mechanisms in reablement	Contextual qualitative interpretation only
Lim et al. (2022) [44]	QuADS	Purposive sample; non-loading participants; limited reflexivity	Q-method role typologies	No population prevalence
Rouch et al. (2022) [45]	JBI qualitative	Small self-selected sample; single-PI analytic influence	Primary-care barriers and facilitators	No frequency estimates
Missen et al. (2021) [46]	JBI quasi-experimental	No concurrent control; staffing changes occurred during implementation	Observed local waiting-list change	Not interpreted as causal effectiveness
Smith & Gallego (2021) [47]	MMAT mixed methods	Small selected parent sample; outreach before survey; limited integration	Access barriers and proof-of-concept service adaptation	No population prevalence or effectiveness
Raymond et al. (2020) [48]	JBI qualitative	Scenario-based judgments and transferability limits	Stakeholder perspectives on prioritization	Not actual referral behavior or frequency
Bolt et al. (2019) [31]	MMAT quantitative descriptive + JBI expert opinion	Limited survey reporting; empirical and advocacy components mixed	Member-association findings and professional recommendations	Survey findings and proposals separated
Dickson & Toto (2018) [49]	JBI quasi-experimental	Small selected single group; no control; short follow-up	Feasibility of OT integration	No effectiveness, cost, or institutionalization conclusion
Friedman & VanPuymbrouck (2018) [50]	MMAT mixed methods	Waiver projections rather than actual use; single qualitative coder	Direct coverage and financing structures	Planned and actual utilization distinguished
Dahl-Popolizio et al. (2017) [51]	MMAT quantitative descriptive	Convenience/snowball sample; unknown response denominator; unvalidated survey	Respondent receptiveness and role perceptions	No representative prevalence
Koverman et al. (2017) [52]	QuADS	Limited sampling, data collection, analytic, ethical, and audit-trail reporting	Implemented workflow, EMR, scheduling, and billing arrangements	No success, referral-effect, or effectiveness claim
Raymond et al. (2016) [53]	JBI analytical cross-sectional	Cross-sectional design; proxy waiting outcome; residual confounding	Waiting-list management patterns and associations	No causal inference
Wilberforce et al. (2016) [54]	JBI analytical cross-sectional	Self-reported time use; measurement validity unclear; one trust	Workforce deployment and role distribution	Limited transferability
Donnelly et al. (2013) [55]	JBI qualitative	Four early-stage Ontario cases; limited reflexivity; no patient outcomes	Organizational integration structures and processes	Not universally necessary or sufficient conditions
Raymond et al. (2013) [56]	JBI analytical cross-sectional	Regional transferability; constructed calibration; companion publication	Prioritization criteria and waiting patterns	Counted with 2016 report as one dataset
London-Willis et al. (2012) [57]	JBI qualitative	Six early volunteers; saturation not reported	Policy-related professional role change	No system-wide generalization
Carrier et al. (2010) [58]	QuADS	Document flow, dual review, and audit trail not reported	Legal and ethical interpretation of access/resource allocation	Not empirical service-effect evidence
Quick et al. (2010) [59]	MMAT quantitative descriptive	Sampling denominator, non-response, and measure validation unclear	Reported scope of practice and barriers	No representative or causal inference

EMR, electronic medical record; JBI, Joanna Briggs Institute; MMAT, Mixed Methods Appraisal Tool; OT, occupational therapy; PI, principal investigator; QuADS, Quality Assessment for Diverse Studies.

**Table 7:** Concise cross-jurisdictional comparison of occupational therapy integration.

Jurisdiction	Main Care Context	Distinctive Policy/System Pattern	Main Barrier/Enabler	Interpretive Boundary
Global/ WFOT	Primary care	Wide variation in formal recognition, financing, coverage, and service models.	Workforce supply, funding, and training repeatedly constrained implementation.	Member-organization survey; high-income settings overrepresented.
United States	Primary care; LTSS care coordination; Medicaid HCBS	Reimbursement, billing, waiver design, referral pathways, and team integration were prominent.	Role recognition, workflow, leadership, and reimbursement shaped implementation.	Mostly local/convenience samples, except for national waiver analysis.
Canada (Quebec/ Ontario)	Home/community OT; Family Health Teams	Waiting-list prioritization and access were prominent in Quebec; shared records, co-location, and team processes supported integration in Ontario.	Resource constraints, fairness, role understanding, and trust influenced delivery.	Mostly regional qualitative/program data; limited downstream outcomes.
United Kingdom	Reablement; integrated health/social care; adult social care	Role redesign and service integration occurred within commissioning and personalization contexts.	Co-location, training, workload distribution, and role identity shaped implementation.	Local/small qualitative evidence plus one single-trust time-use study.
Australia	Community health; rural/community OT	Assessment models, outreach, and intra-disciplinary support addressed local access and service-delivery constraints.	Management support, funding, rural workforce capacity, and transport/health-literacy barriers mattered.	Predominantly descriptive/local evidence; one uncontrolled pre/post evaluation.
South Korea	Home-based rehabilitation	OT roles were framed within a team-based home rehabilitation model.	Collaboration, service reliability, client needs, and professional development were emphasized.	Single small Q-methodology study.
Europe/ COTEC	Primary/community care	Country-to-country variation in organization and financing was reported.	Professional recognition and wider primary-care integration were emphasized.	Limited survey reporting; the position component was normative.

COTEC, Council of Occupational Therapists for the European Countries; HCBS, Home and Community-Based Services; LTSS, Long-Term Services and Supports; OT, occupational therapy; WFOT, World Federation of Occupational Therapists.

**Table 8:** Synthesis of findings by research question with contributing reports included.

Research Question	Synthesized Answer	Interpretive Boundary
RQ1. How is OT positioned in policy and institutional terms?	OT was positioned through primary-care teams, publicly organized home/community services, Medicaid HCBS coverage, LTSS care coordination, reablement/social care, community health, and home-based rehabilitation. Formal recognition varied by jurisdiction and did not itself ensure service availability.	Evidence is primarily descriptive and jurisdiction-specific; institutional presence should not be interpreted as universal coverage or access.
RQ2. What service and system functions are attributed to OT?	Across settings, OT functions included contextualized functional/ADL-IADL assessment, environmental modification, participation-focused intervention, reablement, self-management, caregiver support, assistive technology, coordination, role consultation, and support-worker/team education.	Roles are consistently described, but comparative evidence identifying a superior role configuration is absent.
RQ3. How do financing, coverage, referral, access, and delivery arrangements vary?	Variation was documented in public financing, Medicaid waiver coverage and reimbursement, billing, referral routes, prioritization rules, waiting-list management, outreach, EMR/shared records, staffing, and local service organization.	The evidence demonstrates heterogeneity but does not establish a single optimal financing, referral, or delivery model.
RQ4. Which policy/system outcomes are documented?	Directly reported outcomes concerned waiting time, attendance/access, service activity, projected expenditure/utilisation, workforce time use, and implementation feasibility. Stakeholder studies documented concerns about fairness and access. Hospitalization, institutionalization, cost savings, and long-term care demand were not established as effects of OT integration.	Quantitative outcome evidence was sparse and mostly non-randomized; downstream system effects remain unconfirmed.
RQ5. Which conditions recur in implementation?	Five recurrent policy-system domains were supported across the evidence: institutional recognition; financing/coverage; referral/coordination; service-delivery infrastructure; and workforce/implementation support. Access, equity, and user participation cut across these domains.	These domains represent higher-order organizing categories refined from heterogeneous evidence and should be interpreted in relation to the contexts of the contributing studies.

ADL, activities of daily living; EMR, electronic medical record; HCBS, Home and Community-Based Services; IADL, instrumental activities of daily living; LTSS, Long-Term Services and Supports; OT, occupational therapy; RQ1–RQ5, research questions 1–5.

**Table 9:** Summary of sensitivity analyses and their interpretive implications.

Sensitivity Restriction	Result	Interpretive Consequence
Exclude professional/normative recommendations	All five organizing domains remained identifiable.	The framework was not dependent on advocacy statements.
Restrict synthesis to directly reported findings and remove unmeasured downstream outcomes	All five domains remained; claims about hospitalization, institutionalization, costs, and long-term care demand were removed or reframed as hypotheses.	Direct system arrangements could be described, but downstream effects could not be claimed.
Control companion publications and restrict structured implementation reports to traceable operational evidence	Core referral/access and implementation patterns remained.	Double-counting and unsupported implementation claims were reduced.
Restrict uncontrolled studies to feasibility or observed local change	Access and implementation patterns remained, but causal-effect language was removed.	Uncontrolled studies informed feasibility/local change only.

**Table 10:** Summary of confidence in qualitative findings and certainty of quantitative outcomes.

Evidence Finding/Outcome	Approach	Rating	Interpretation in This Review
Role clarity and team understanding	GRADE-CERQual	Moderate	Consistent pattern, but some limitations in data richness/context breadth.
Co-location, shared records, team meetings, and managerial support	GRADE-CERQual	Moderate	Supports organizational integration, with some contextual limitations.
Service context and workforce capacity shape OT roles	GRADE-CERQual	Moderate	Recurrent across settings, but largely in high-income systems.
Reimbursement/billing constraints impede integration	GRADE-CERQual	Low	Limited qualitative data; interpret as a plausible barrier rather than a universal conclusion.
Stakeholders differ in referral-prioritisation/fairness judgments	GRADE-CERQual	Low	Supported by a small evidence base; avoid broad generalization.
Service redesign may create tension with occupation-focused practice	GRADE-CERQual	Low	Limited direct qualitative evidence; use cautiously.
Waiting-time change; waiting-list association; OT-in-care-coordination feasibility	GRADE	Very low (all 3 outcomes)	Observed changes/associations may be reported, but causal effectiveness cannot be established.

CERQual, Confidence in the Evidence from Reviews of Qualitative research; GRADE, Grading of Recommendations Assessment, Development and Evaluation; OT, occupational therapy.

**Table 11:** Integrated policy-system domains refined through framework synthesis of the included evidence.

Synthesized Domain	Pattern Identified Across the Evidence	OT-Related System Contribution	Boundary/Remaining Uncertainty
Policy recognition and institutional positioning	The way in which OT was formally positioned within teams, service specifications, coverage rules, and organizational roles varied across jurisdictions and service systems.	Linking function, occupation, participation, and person–environment fit was recurrently identified as an important OT-related contribution within community-based systems.	Descriptive and observational evidence does not establish that formal recognition itself improves access to services or outcomes.
Financing and coverage	Government funding, Medicaid waiver arrangements, reimbursement and billing mechanisms, and local financing structures were reported as conditions that could enable or constrain the availability and sustainability of OT services.	Financing and coverage arrangements that support community-based functional assessment and intervention were identified as important institutional conditions for service delivery.	Comparative evidence is insufficient to determine which financing or coverage model is most effective or equitable.
Referral and coordination	Referral pathways, triage and prioritization, shared records, team communication, and inter-service linkages were recurrently identified as operational conditions related to timeliness and service integration.	OT contributed information on functional risk, home and environmental conditions, and participation priorities to referral and coordination processes.	The effects of standardized referral and coordination pathways on equity, continuity, and longer-term outcomes remain insufficiently evaluated.
Service-delivery roles and infrastructure	OT roles were reported alongside operational infrastructure, including workflows, access to records, co-location, outreach, and service-delivery models.	Home and environmental assessment, reablement, ADL/IADL, assistive technology, self-management, and caregiver support were recurrent service functions explicitly associated with OT.	Comparative effectiveness across service models, resource requirements, and longer-term system outcomes remains poorly established.
Workforce capacity and implementation support	Workforce supply, role understanding, leadership, managerial support, peer consultation, training, and protected time for team collaboration were reported as facilitators or constraints to OT integration.	OT integration appeared to involve not only workforce availability but also role design, team processes, and organizational support for implementation.	Most evidence was local or self-reported, and transferable workforce or competency standards have not been established.
Cross-cutting considerations: accessibility, equity, and user participation	Waiting time, geographic access, transportation, health literacy, prioritization rules, and differences between professional and user priorities appeared across multiple policy-system domains.	The evidence highlighted the relevance of accessibility, fairness, and community-user priorities to service design and resource allocation.	Evidence from service users and caregivers, rural and remote populations, and diverse cultural and socioeconomic groups remains limited.

ADL, activities of daily living; IADL, instrumental activities of daily living; OT, occupational therapy.

## Data Availability

Final search strategies, the operational eligibility codebook, detailed extraction matrices, item-level critical appraisal results, framework-coding matrices, cross-jurisdictional comparisons, sensitivity analyses, GRADE-CERQual profiles, and GRADE assessments are available in the Appendix A, Appendix B, Appendix C, Appendix D, Appendix E, Appendix F, Appendix G and Appendix H. The methodological record for this review is available in the Open Science Framework (OSF) at https://doi.org/10.17605/OSF.IO/P7WYD. Additional details on the search, study selection, data extraction, and appraisal procedures are available from the author upon reasonable request. No additional supplementary materials are submitted at this stage.

## Appendix A. Full Database-Specific Search Strategies

**Table:**

Database	Complete Search String
PubMed/MEDLINE	(“Occupational Therapy”[MeSH Terms] OR “Occupational Therapists”[MeSH Terms] OR “occupational therapy”[Title/Abstract] OR “occupational therapist”[Title/Abstract] OR “occupational therapy practitioner”[Title/Abstract]) AND (“Community Health Services”[MeSH Terms] OR “Home Care Services”[MeSH Terms] OR “Primary Health Care”[MeSH Terms] OR “Delivery of Health Care, Integrated”[MeSH Terms] OR “Patient-Centered Care”[MeSH Terms] OR “Continuity of Patient Care”[MeSH Terms] OR “Long-Term Care”[MeSH Terms] OR “integrated community care”[Title/Abstract] OR “community-based integrated care”[Title/Abstract] OR “community based integrated care”[Title/Abstract] OR “integrated health and social care”[Title/Abstract] OR “integrated care”[Title/Abstract] OR “integrated health care”[Title/Abstract] OR “integrated healthcare”[Title/Abstract] OR “care integration”[Title/Abstract] OR “integrated service delivery”[Title/Abstract] OR “community care”[Title/Abstract] OR “community-based care”[Title/Abstract] OR “community based care”[Title/Abstract] OR “home care”[Title/Abstract] OR “home health care”[Title/Abstract] OR “home-based care”[Title/Abstract] OR “home based care”[Title/Abstract] OR “home and community-based service*”[Title/Abstract] OR “home and community based service*”[Title/Abstract] OR HCBS[Title/Abstract] OR “primary care”[Title/Abstract] OR “primary health care”[Title/Abstract] OR “community health service*”[Title/Abstract] OR “community support service*”[Title/Abstract] OR “community rehabilitation”[Title/Abstract] OR “community-based rehabilitation”[Title/Abstract] OR “community based rehabilitation”[Title/Abstract] OR “home-based rehabilitation”[Title/Abstract] OR “home based rehabilitation”[Title/Abstract] OR “ambulatory rehabilitation”[Title/Abstract] OR “social care”[Title/Abstract] OR “long-term services and supports”[Title/Abstract] OR “long term services and supports”[Title/Abstract] OR LTSS[Title/Abstract] OR “long-term care”[Title/Abstract] OR “long term care”[Title/Abstract] OR reablement[Title/Abstract] OR “care transition*”[Title/Abstract] OR “transitional care”[Title/Abstract] OR “continuity of care”[Title/Abstract] OR “aging in place”[Title/Abstract] OR “ageing in place”[Title/Abstract]) AND (“Health Policy”[MeSH Terms] OR “Public Policy”[MeSH Terms] OR “Policy Making”[MeSH Terms] OR “Health Services Administration”[MeSH Terms] OR “Delivery of Health Care”[MeSH Terms] OR “Health Services Accessibility”[MeSH Terms] OR “Referral and Consultation”[MeSH Terms] OR “Case Management”[MeSH Terms] OR “Patient Care Team”[MeSH Terms] OR “Interprofessional Relations”[MeSH Terms] OR “Health Workforce”[MeSH Terms] OR “Health Care Reform”[MeSH Terms] OR “Insurance Coverage”[MeSH Terms] OR “Reimbursement Mechanisms”[MeSH Terms] OR “Financing, Organized”[MeSH Terms] OR “Health Planning”[MeSH Terms] OR “Models, Organizational”[MeSH Terms] OR “Resource Allocation”[MeSH Terms] OR “health polic*”[Title/Abstract] OR “public polic*”[Title/Abstract] OR policy[Title/Abstract] OR policies[Title/Abstract] OR policymak*[Title/Abstract] OR “policy making”[Title/Abstract] OR “policy development”[Title/Abstract] OR “healthcare policy”[Title/Abstract] OR “health care policy”[Title/Abstract] OR “health service*”[Title/Abstract] OR “health services research”[Title/Abstract] OR “service deliver*”[Title/Abstract] OR “service organization”[Title/Abstract] OR “service organisation”[Title/Abstract] OR “service structur*”[Title/Abstract] OR “service model*”[Title/Abstract] OR “care model*”[Title/Abstract] OR “model* of care”[Title/Abstract] OR “delivery model*”[Title/Abstract] OR governance[Title/Abstract] OR legislat*[Title/Abstract] OR regulat*[Title/Abstract] OR financ*[Title/Abstract] OR funding[Title/Abstract] OR reimburse*[Title/Abstract] OR payment*[Title/Abstract] OR coverage[Title/Abstract] OR “insurance coverage”[Title/Abstract] OR “service coverage”[Title/Abstract] OR eligib*[Title/Abstract] OR entitlement*[Title/Abstract] OR “benefit structur*”[Title/Abstract] OR “benefit design”[Title/Abstract] OR “benefit package*”[Title/Abstract] OR referral*[Title/Abstract] OR “referral pathway*”[Title/Abstract] OR “care pathway*”[Title/Abstract] OR “care coordination”[Title/Abstract] OR “service coordination”[Title/Abstract] OR “case management”[Title/Abstract] OR commission*[Title/Abstract] OR contract*[Title/Abstract] OR procur*[Title/Abstract] OR workforce[Title/Abstract] OR staffing[Title/Abstract] OR “workforce capacity”[Title/Abstract] OR “workforce deployment”[Title/Abstract] OR “workforce planning”[Title/Abstract] OR “workforce distribution”[Title/Abstract] OR “human resources for health”[Title/Abstract] OR implement*[Title/Abstract] OR “implementation condition*”[Title/Abstract] OR “implementation strateg*”[Title/Abstract] OR “health system reform*”[Title/Abstract] OR “health care reform*”[Title/Abstract] OR “healthcare reform*”[Title/Abstract] OR “organizational change”[Title/Abstract] OR “organisational change”[Title/Abstract] OR “service redesign”[Title/Abstract] OR “system redesign”[Title/Abstract] OR “health system redesign”[Title/Abstract] OR “service access”[Title/Abstract] OR “service accessibility”[Title/Abstract] OR accessibility[Title/Abstract] OR accessible[Title/Abstract] OR “access to care”[Title/Abstract] OR “access to services”[Title/Abstract] OR availab*[Title/Abstract] OR “waiting list*”[Title/Abstract] OR “wait list*”[Title/Abstract] OR “waiting time*”[Title/Abstract] OR “wait time*”[Title/Abstract] OR “resource allocation”[Title/Abstract] OR “interprofessional care”[Title/Abstract] OR “interdisciplinary care”[Title/Abstract] OR “multidisciplinary care”[Title/Abstract] OR “team-based care”[Title/Abstract] OR “team based care”[Title/Abstract] OR “collaborative care”[Title/Abstract] OR “professional role*”[Title/Abstract] OR “role delineation”[Title/Abstract] OR “role clarity”[Title/Abstract] OR “scope of practice”[Title/Abstract] OR “institutional position*”[Title/Abstract] OR “institutional recognition”[Title/Abstract] OR “formal recognition”[Title/Abstract] OR “professional position*”[Title/Abstract] OR “role integration”[Title/Abstract] OR “professional integration”[Title/Abstract])
CINAHL	(MH “Occupational Therapy+” OR TI (“occupational therapy” OR “occupational therapist*” OR “occupational therapy practitioner*”) OR AB (“occupational therapy” OR “occupational therapist*” OR “occupational therapy practitioner*”)) AND (MH “Community Health Services+” OR MH “Home Health Care+” OR MH “Primary Health Care” OR MH “Health Care Delivery, Integrated” OR MH “Continuity of Patient Care+” OR MH “Long Term Care+” OR TI (“integrated community care” OR “community-based integrated care” OR “community based integrated care” OR “integrated health and social care” OR “integrated care” OR “integrated health care” OR “integrated healthcare” OR “care integration” OR “integrated service delivery” OR “community care” OR “community-based care” OR “community based care” OR “home care” OR “home health care” OR “home-based care” OR “home based care” OR “home and community-based service*” OR “home and community based service*” OR HCBS OR “primary care” OR “primary health care” OR “community health service*” OR “community support service*” OR “community rehabilitation” OR “community-based rehabilitation” OR “community based rehabilitation” OR “home-based rehabilitation” OR “home based rehabilitation” OR “ambulatory rehabilitation” OR “social care” OR “long-term services and supports” OR “long term services and supports” OR LTSS OR “long-term care” OR “long term care” OR reablement OR “care transition*” OR “transitional care” OR “continuity of care” OR “aging in place” OR “ageing in place”) OR AB (“integrated community care” OR “community-based integrated care” OR “community based integrated care” OR “integrated health and social care” OR “integrated care” OR “integrated health care” OR “integrated healthcare” OR “care integration” OR “integrated service delivery” OR “community care” OR “community-based care” OR “community based care” OR “home care” OR “home health care” OR “home-based care” OR “home based care” OR “home and community-based service*” OR “home and community based service*” OR HCBS OR “primary care” OR “primary health care” OR “community health service*” OR “community support service*” OR “community rehabilitation” OR “community-based rehabilitation” OR “community based rehabilitation” OR “home-based rehabilitation” OR “home based rehabilitation” OR “ambulatory rehabilitation” OR “social care” OR “long-term services and supports” OR “long term services and supports” OR LTSS OR “long-term care” OR “long term care” OR reablement OR “care transition*” OR “transitional care” OR “continuity of care” OR “aging in place” OR “ageing in place”)) AND (MH “Health Policy+” OR MH “Public Policy+” OR MH “Policy Making” OR MH “Health Services Administration+” OR MH “Health Services Accessibility+” OR MH “Referral and Consultation+” OR MH “Case Management” OR MH “Multidisciplinary Care Team+” OR MH “Interprofessional Relations” OR MH “Health Care Reform” OR MH “Insurance Coverage+” OR MH “Reimbursement Mechanisms+” OR MH “Health Planning+” OR MH “Resource Allocation+” OR TI (“health polic*” OR “public polic*” OR policy OR policies OR policymak* OR “policy making” OR “policy development” OR “healthcare policy” OR “health care policy” OR “health service*” OR “health services research” OR “service deliver*” OR “service organization” OR “service organisation” OR “service structur*” OR “service model*” OR “care model*” OR “model* of care” OR “delivery model*” OR governance OR legislat* OR regulat* OR financ* OR funding OR reimburse* OR payment* OR coverage OR “insurance coverage” OR “service coverage” OR eligib* OR entitlement* OR “benefit structur*” OR “benefit design” OR “benefit package*” OR referral* OR “referral pathway*” OR “care pathway*” OR “care coordination” OR “service coordination” OR “case management” OR commission* OR contract* OR procur* OR workforce OR staffing OR “workforce capacity” OR “workforce deployment” OR “workforce planning” OR “workforce distribution” OR “human resources for health” OR implement* OR “implementation condition*” OR “implementation strateg*” OR “health system reform*” OR “health care reform*” OR “healthcare reform*” OR “organizational change” OR “organisational change” OR “service redesign” OR “system redesign” OR “health system redesign” OR “service access” OR “service accessibility” OR accessibility OR accessible OR “access to care” OR “access to services” OR availab* OR “waiting list*” OR “wait list*” OR “waiting time*” OR “wait time*” OR “resource allocation” OR “interprofessional care” OR “interdisciplinary care” OR “multidisciplinary care” OR “team-based care” OR “team based care” OR “collaborative care” OR “professional role*” OR “role delineation” OR “role clarity” OR “scope of practice” OR “institutional position*” OR “institutional recognition” OR “formal recognition” OR “professional position*” OR “role integration” OR “professional integration”) OR AB (“health polic*” OR “public polic*” OR policy OR policies OR policymak* OR “policy making” OR “policy development” OR “healthcare policy” OR “health care policy” OR “health service*” OR “health services research” OR “service deliver*” OR “service organization” OR “service organisation” OR “service structur*” OR “service model*” OR “care model*” OR “model* of care” OR “delivery model*” OR governance OR legislat* OR regulat* OR financ* OR funding OR reimburse* OR payment* OR coverage OR “insurance coverage” OR “service coverage” OR eligib* OR entitlement* OR “benefit structur*” OR “benefit design” OR “benefit package*” OR referral* OR “referral pathway*” OR “care pathway*” OR “care coordination” OR “service coordination” OR “case management” OR commission* OR contract* OR procur* OR workforce OR staffing OR “workforce capacity” OR “workforce deployment” OR “workforce planning” OR “workforce distribution” OR “human resources for health” OR implement* OR “implementation condition*” OR “implementation strateg*” OR “health system reform*” OR “health care reform*” OR “healthcare reform*” OR “organizational change” OR “organisational change” OR “service redesign” OR “system redesign” OR “health system redesign” OR “service access” OR “service accessibility” OR accessibility OR accessible OR “access to care” OR “access to services” OR availab* OR “waiting list*” OR “wait list*” OR “waiting time*” OR “wait time*” OR “resource allocation” OR “interprofessional care” OR “interdisciplinary care” OR “multidisciplinary care” OR “team-based care” OR “team based care” OR “collaborative care” OR “professional role*” OR “role delineation” OR “role clarity” OR “scope of practice” OR “institutional position*” OR “institutional recognition” OR “formal recognition” OR “professional position*” OR “role integration” OR “professional integration”))
Scopus	TITLE-ABS-KEY(“occupational therapy” OR “occupational therapist*” OR “occupational therapy practitioner*”) AND TITLE-ABS-KEY(“integrated community care” OR “community-based integrated care” OR “community based integrated care” OR “integrated health and social care” OR “integrated care” OR “integrated health care” OR “integrated healthcare” OR “care integration” OR “integrated service delivery” OR “community care” OR “community-based care” OR “community based care” OR “home care” OR “home health care” OR “home-based care” OR “home based care” OR “home and community-based service*” OR “home and community based service*” OR HCBS OR “primary care” OR “primary health care” OR “community health service*” OR “community support service*” OR “community rehabilitation” OR “community-based rehabilitation” OR “community based rehabilitation” OR “home-based rehabilitation” OR “home based rehabilitation” OR “ambulatory rehabilitation” OR “social care” OR “long-term services and supports” OR “long term services and supports” OR LTSS OR “long-term care” OR “long term care” OR reablement OR “care transition*” OR “transitional care” OR “continuity of care” OR “aging in place” OR “ageing in place”) AND TITLE-ABS-KEY(“health polic*” OR “public polic*” OR policy OR policies OR policymak* OR “policy making” OR “policy development” OR “healthcare policy” OR “health care policy” OR “health service*” OR “health services research” OR “service deliver*” OR “service organization” OR “service organisation” OR “service structur*” OR “service model*” OR “care model*” OR “model* of care” OR “delivery model*” OR governance OR legislat* OR regulat* OR financ* OR funding OR reimburse* OR payment* OR coverage OR “insurance coverage” OR “service coverage” OR eligib* OR entitlement* OR “benefit structur*” OR “benefit design” OR “benefit package*” OR referral* OR “referral pathway*” OR “care pathway*” OR “care coordination” OR “service coordination” OR “case management” OR commission* OR contract* OR procur* OR workforce OR staffing OR “workforce capacity” OR “workforce deployment” OR “workforce planning” OR “workforce distribution” OR “human resources for health” OR implement* OR “implementation condition*” OR “implementation strateg*” OR “health system reform*” OR “health care reform*” OR “healthcare reform*” OR “organizational change” OR “organisational change” OR “service redesign” OR “system redesign” OR “health system redesign” OR “service access” OR “service accessibility” OR accessibility OR accessible OR “access to care” OR “access to services” OR availab* OR “waiting list*” OR “wait list*” OR “waiting time*” OR “wait time*” OR “resource allocation” OR “interprofessional care” OR “interdisciplinary care” OR “multidisciplinary care” OR “team-based care” OR “team based care” OR “collaborative care” OR “professional role*” OR “role delineation” OR “role clarity” OR “scope of practice” OR “institutional position*” OR “institutional recognition” OR “formal recognition” OR “professional position*” OR “role integration” OR “professional integration”)
ProQuest Central	NOFT(((“occupational therapy” OR “occupational therapist*” OR “occupational therapy practitioner*”) AND (“integrated community care” OR “community-based integrated care” OR “community based integrated care” OR “integrated health and social care” OR “integrated care” OR “integrated health care” OR “integrated healthcare” OR “care integration” OR “integrated service delivery” OR “community care” OR “community-based care” OR “community based care” OR “home care” OR “home health care” OR “home-based care” OR “home based care” OR “home and community-based service*” OR “home and community based service*” OR HCBS OR “primary care” OR “primary health care” OR “community health service*” OR “community support service*” OR “community rehabilitation” OR “community-based rehabilitation” OR “community based rehabilitation” OR “home-based rehabilitation” OR “home based rehabilitation” OR “ambulatory rehabilitation” OR “social care” OR “long-term services and supports” OR “long term services and supports” OR LTSS OR “long-term care” OR “long term care” OR reablement OR “care transition*” OR “transitional care” OR “continuity of care” OR “aging in place” OR “ageing in place” OR “home and community care” OR “community service*” OR “community mental health”) AND (“health polic*” OR “public polic*” OR policy OR policies OR policymak* OR “policy making” OR “policy development” OR “healthcare policy” OR “health care policy” OR “health service*” OR “health services research” OR “service deliver*” OR “service organization” OR “service organisation” OR “service structur*” OR “service model*” OR “care model*” OR “model* of care” OR “delivery model*” OR governance OR legislat* OR regulat* OR financ* OR funding OR reimburse* OR payment* OR coverage OR “insurance coverage” OR “service coverage” OR eligib* OR entitlement* OR “benefit structur*” OR “benefit design” OR “benefit package*” OR referral* OR “referral pathway*” OR “care pathway*” OR “care coordination” OR “service coordination” OR “case management” OR commission* OR contract* OR procur* OR workforce OR staffing OR “workforce capacity” OR “workforce deployment” OR “workforce planning” OR “workforce distribution” OR “human resources for health” OR implement* OR “implementation condition*” OR “implementation strateg*” OR “health system reform*” OR “health care reform*” OR “healthcare reform*” OR “organizational change” OR “organisational change” OR “service redesign” OR “system redesign” OR “health system redesign” OR “service access” OR “service accessibility” OR accessibility OR accessible OR “access to care” OR “access to services” OR availab* OR “waiting list*” OR “wait list*” OR “waiting time*” OR “wait time*” OR “resource allocation” OR “interprofessional care” OR “interdisciplinary care” OR “multidisciplinary care” OR “team-based care” OR “team based care” OR “collaborative care” OR “professional role*” OR “role delineation” OR “role clarity” OR “scope of practice” OR “institutional position*” OR “institutional recognition” OR “formal recognition” OR “professional position*” OR “role integration” OR “professional integration”)))
APA PsycINFO	(“occupational therapy” OR “occupational therapist*” OR “occupational therapy practitioner*”) AND (“integrated community care” OR “community-based integrated care” OR “community based integrated care” OR “integrated health and social care” OR “integrated care” OR “integrated health care” OR “integrated healthcare” OR “care integration” OR “integrated service delivery” OR “community care” OR “community-based care” OR “community based care” OR “home care” OR “home health care” OR “home-based care” OR “home based care” OR “home and community-based service*” OR “home and community based service*” OR HCBS OR “primary care” OR “primary health care” OR “community health service*” OR “community support service*” OR “community rehabilitation” OR “community-based rehabilitation” OR “community based rehabilitation” OR “home-based rehabilitation” OR “home based rehabilitation” OR “ambulatory rehabilitation” OR “social care” OR “long-term services and supports” OR “long term services and supports” OR LTSS OR “long-term care” OR “long term care” OR reablement OR “care transition*” OR “transitional care” OR “continuity of care” OR “aging in place” OR “ageing in place” OR “service integration” OR “integrated service*” OR “rehabilitation service*” OR “intermediate care”) AND (“health polic*” OR “public polic*” OR policy OR policies OR policymak* OR “policy making” OR “policy development” OR “healthcare policy” OR “health care policy” OR “health service*” OR “health services research” OR “service deliver*” OR “service organization” OR “service organisation” OR “service structur*” OR “service model*” OR “care model*” OR “model* of care” OR “delivery model*” OR governance OR legislat* OR regulat* OR financ* OR funding OR reimburse* OR payment* OR coverage OR “insurance coverage” OR “service coverage” OR eligib* OR entitlement* OR “benefit structur*” OR “benefit design” OR “benefit package*” OR referral* OR “referral pathway*” OR “care pathway*” OR “care coordination” OR “service coordination” OR “case management” OR commission* OR contract* OR procur* OR workforce OR staffing OR “workforce capacity” OR “workforce deployment” OR “workforce planning” OR “workforce distribution” OR “human resources for health” OR implement* OR “implementation condition*” OR “implementation strateg*” OR “health system reform*” OR “health care reform*” OR “healthcare reform*” OR “organizational change” OR “organisational change” OR “service redesign” OR “system redesign” OR “health system redesign” OR “service access” OR “service accessibility” OR accessibility OR accessible OR “access to care” OR “access to services” OR availab* OR “waiting list*” OR “wait list*” OR “waiting time*” OR “wait time*” OR “resource allocation” OR “interprofessional care” OR “interdisciplinary care” OR “multidisciplinary care” OR “team-based care” OR “team based care” OR “collaborative care” OR “professional role*” OR “role delineation” OR “role clarity” OR “scope of practice” OR “institutional position*” OR “institutional recognition” OR “formal recognition” OR “professional position*” OR “role integration” OR “professional integration”)

* indicates a truncation wildcard used to retrieve terms with variant word endings (e.g., therapist and therapists). AB, abstract field; APA, American Psychological Association; CINAHL, Cumulative Index to Nursing and Allied Health Literature; HCBS, Home and Community-Based Services; LTSS, Long-Term Services and Supports; MEDLINE, Medical Literature Analysis and Retrieval System Online; MeSH, Medical Subject Headings; MH, subject-heading field; NOFT, anywhere except full text (ProQuest field); TI, title field; TITLE-ABS-KEY, title, abstract, and keywords field.

## Appendix B. Operational Eligibility Codebook Used by Both Reviewers

**Table:**

Eligibility Domain	Decision	Operational Includes Rule	Operational Exclude Rule	Reviewer Instruction
Context/community setting	Include	Primary care, community health, home care/home health, home-based rehabilitation, reablement, social care, HCBS/LTSS, or hospital-to-community transition with an explicit community component.	Exclude hospital-, inpatient-, residential- or institution-only care without a separable community pathway.	If eligibility was unclear during independent screening, the record was retained for reconciliation. During reconciliation, exclusion required agreement between both reviewers that the available title and abstract information clearly failed to meet at least one mandatory eligibility criterion; records that remained uncertain or discrepant were advanced to full-text assessment.
Occupational therapy relevance	Include	OT service, therapist, workforce, or OT-related function is explicit, and at least one OT-related finding can be extracted.	Exclude studies where OT is absent, incidental, only listed among professions, or inseparable from generic multidisciplinary rehabilitation.	Require an OT-related extractable result, not just a background mention.
Policy/system relevance	Include	At least one substantive result on institutional position, eligibility, financing/coverage/reimbursement, referral/coordination, access/waiting, service organization, workforce, governance, or implementation.	Exclude papers in which policy/system terms occur only in the introduction/discussion or that report only individual clinical effectiveness.	The relevant result must be part of the study findings or documented service/policy analysis.
Study design/analytic basis	Include	Original qualitative, quantitative, mixed-methods, health-services, organizational, workforce, implementation, comparative, or analytical document/legal studies; structured implementation/service-development reports with implemented service, identifiable operational data, and traceable OT-related findings.	Exclude reviews, editorials, commentaries, conference abstracts, protocols, purely promotional/advocacy reports without an identifiable analytic or operational source base.	For implementation reports, verify actual implementation plus traceable operational data.
Publication type	Include	Peer-reviewed full journal article.	Exclude grey literature, association webpages/reports, dissertations, conference abstracts/proceedings, preprints, and non-peer-reviewed reports.	Peer-review status must be identifiable.
Language	Include	English.	Exclude non-English full text.	Apply after bibliographic identification.
Publication period	Include	1 January 2010 to 29 July 2026.	Exclude before 1 January 2010 or after 29 July 2026.	Use the publication date of the final journal version.
Full-text availability	Include	The complete article is available for eligibility and data extraction.	Exclude when the complete text cannot be obtained after reasonable retrieval attempts.	Exclusion for unavailable full text was applied only after reasonable efforts to retrieve the full text had been made.
Companion publications	Include as separate reports only if distinct questions/outcomes	Retain multiple reports from one project when they address substantively distinct review questions or outcomes.	Do not count companion reports as independent datasets when judging evidence breadth.	Link shared samples/datasets explicitly; companion reports derived from the same underlying dataset should be treated as a single dataset when judging evidence breadth.
Decision hierarchy	Apply one primary reason for exclusion at full-text assessment.	When several criteria fail, record the first decisive reason in this order: publication type → date/language/full text → context → OT relevance → policy/system relevance → design/analytic basis.	Do not assign multiple primary reasons to one full-text exclusion.	Use identical hierarchy for both reviewers.
Title/abstract reconciliation	Re-examine	Records retained by at least one reviewer were re-examined against the same eligibility codebook at the title/abstract level.	Exclude a record only when both reviewers agree, after re-examination, that at least one mandatory eligibility criterion is clearly not met.	Apply the same rules as the initial screening; this was not a full-text assessment.

HCBS, Home and Community-Based Services; LTSS, Long-Term Services and Supports; OT, occupational therapy.

## Appendix C. Detailed Extraction Matrix for the 21 Included Reports

**Table:**

Study	Country/ Jurisdiction	Care Context	Design/Sample or Data Source	OT-Related Focus	Primary Policy/System Domain
Freeman et al. (2025) [12]	Global/ WFOT	Primary care	Cross-sectional survey; 59/100 WFOT member organizations	Global mapping of OT primary-care services, settings, populations, financing, workforce and enabling factors	Policy/institutional position; financing; workforce
Nash et al. (2025) [41]	United States	Primary care	Mixed methods; 41 PCPs, 305 OTs; interviews with 6 PCPs and 1 OT	Receptiveness to OT inclusion, delegation preferences, reimbursement, and role barriers	Institutional position; financing; workforce
Evans et al. (2023) [42]	Australia	Community health	Cross-sectional mixed-methods survey; 80 OTs	Intra-disciplinary OT practice, peer consultation, management support, funding, and organizational barriers	Service delivery; workforce
Dibsdall (2022) [43]	United Kingdom	Reablement/social care	Realist qualitative multiple-case study; 3 reablement services	OT assessment, goal setting, intervention selection, support-worker training, co-location and communication	Service delivery; implementation
Lim et al. (2022) [44]	South Korea	Home-based rehabilitation	Q methodology; 34 rehabilitation-team members	Five role-perception types emphasizing home adaptation, participation, service reliability, client needs, and professional development	Institutional position; service delivery; workforce
Rouch et al. (2022) [45]	United States	Primary care	Descriptive qualitative study; 7 OTs	Entry to primary-care teams, team navigation, patient-centered practice, reimbursement and expansion barriers	Institutional position; financing; referral; workforce
Missen et al. (2021) [46]	Australia	Rural community health	Pre/post service evaluation; 456 clients	Basic Assessment Model using OT/AHA workflow; median wait decreased from 105 to 80 days	Access/waiting; service delivery; workforce
Smith & Gallego (2021) [47]	Australia	Community child health	Mixed methods: routine service data plus parent/carer survey (*n* = 21)	Outreach, attendance, and transport/health-literacy barriers in a disadvantaged area	Access/equity; service delivery
Raymond et al. (2020) [48]	Canada/ Quebec	Home-care OT	Qualitative component: 11 OTs, 10 older persons, 9 adults with disabilities	Stakeholder differences in referral-prioritization rationales: safety, independence and social participation	Referral/prioritisation; equity; participation
Bolt et al. (2019) [31]	Europe/ COTEC	Primary/community care	Survey of 20/30 COTEC member associations embedded in a position paper	Cross-country service/funding organization plus professional recommendations for primary-care positioning	Institutional position; financing; service delivery
Dickson & Toto (2018) [49]	United States	LTSS care coordination	Single-group feasibility study; 23 referred, 16 evaluated	Feasibility of embedding OT evaluation and goal-based intervention in an aging-in-place care-coordination program	Service delivery; coordination; implementation
Friedman & VanPuymbrouck (2018) [50]	United States	Medicaid HCBS/LTSS	Mixed quantitative/document analysis; 111 waivers from 46 states and DC	OT waiver coverage, projected utilization, expenditures, reimbursement, and service definitions	Financing/coverage; policy design
Dahl-Popolizio et al. (2017) [51]	United States	Primary care	Descriptive survey with open-ended responses; OTs and PCPs	Receptiveness to OT on primary-care teams and perceived funding/role barriers	Institutional position; service delivery; financing
Koverman et al. (2017) [52]	United States	Geriatric primary care	Structured implementation/service-development report; 8-week pilot	Operational development of OT service using logic model, EMR, scheduling, referral, and reimbursement processes	Service infrastructure; financing; implementation
Raymond et al. (2016) [53]	Canada/ Quebec	Home-care OT	Cross-sectional program survey; 55 programs	Waiting-list management strategies for low-priority clients and association with wait-list length	Access/waiting; service management
Wilberforce et al. (2016) [54]	United Kingdom	Integrated health and social care	Cross-sectional time-use pilot; 151 practitioners	Distribution of OT and assistant time across direct care, indirect casework, team/service activity, and therapeutic work	Workforce; service organization
Donnelly et al. (2013) [55]	Canada/ Ontario	Primary care	Multiple case studies; 4 Family Health Teams	Organizational structures supporting OT integration: communication, trust, role understanding, co-location, EMR, and team meetings	Institutional position; referral/coordination; service delivery/infrastructure; workforce/implementation
Raymond et al. (2013) [56]	Canada/ Quebec	Home-care OT	Survey/content analysis; 55 programs	Referral-prioritization criteria and wide differences in wait times by priority level	Referral/prioritisation; access/equity
London-Willis et al. (2012) [57]	United Kingdom	Adult social care	Qualitative interviews; 6 OTs	Perceived effects of personalization on OT roles, identity, opportunities and threats	Institutional position; workforce; service delivery
Carrier et al. (2010) [58]	Canada/ Quebec	Community OT services	Legal, ethical and clinical document analysis	Access, prioritization, waiting, resource allocation, distributive justice and service quality	Financing/coverage; referral/coordination
Quick et al. (2010) [59]	Australia/ Victoria	Community health	Descriptive postal survey; 101 returned, 72 eligible	Scope of practice, health promotion, funding/resource barriers, and workforce characteristics	Service delivery; workforce; financing

AHA, Allied Health Assistant; COTEC, Council of Occupational Therapists for the European Countries; DC, District of Columbia; EMR, electronic medical record; HCBS, Home and Community-Based Services; LTSS, Long-Term Services and Supports; OT, occupational therapy; OTs, occupational therapists; PCPs, primary care providers; WFOT, World Federation of Occupational Therapists.

## Appendix D. Study-Level Framework-Synthesis Audit Trail from Provisional Coding to Final Domains

**Table:**

Study	Initial Category Code(s)	Directly Extracted Policy/System Finding	Evidence Status Used in Synthesis	Final Domain Mapping	Cross-Cutting Consideration	Transformation/Decision Rationale
Freeman et al. (2025) [12]	Policy recognition; financing/coverage; workforce/implementation	Global mapping of OT primary-care positioning, financing, workforce and enabling factors	Direct descriptive survey	Policy recognition/institutional positioning; financing/coverage; workforce capacity/implementation support	None	Each arrangement was directly documented and retained.
Nash et al. (2025) [41]	Policy recognition; financing/coverage; workforce/implementation; governance/collaboration	Receptiveness to OT inclusion, delegation preferences, reimbursement, and role barriers	Mixed-method perceptions	Policy recognition/institutional positioning; financing/coverage; workforce capacity/implementation support	None	Governance/collaboration was absorbed into positioning and implementation because it concerned team authority and implementation conditions.
Evans et al. (2023) [42]	Service delivery; workforce/implementation; governance/collaboration	Intra-disciplinary practice, peer consultation, management support, funding, and organizational barriers	Mixed-method descriptive findings	Service-delivery roles/infrastructure; workforce capacity/implementation support	None	Management/collaboration was treated as implementation support rather than a separate governance domain.
Dibsdall (2022) [43]	Service delivery; workforce/implementation; governance/collaboration	Assessment, goal setting, intervention selection, support-worker training, co-location and communication	Qualitative/realist findings	Service-delivery roles/infrastructure; workforce capacity/implementation support	User participation	Co-location/communication mapped to infrastructure/implementation; goal setting informed participation.
Lim et al. (2022) [44]	Policy recognition; service delivery; workforce/implementation	Role-perception types related to home adaptation, participation, service reliability, client needs, and professional development	Q-methodology findings	Policy recognition/institutional positioning; service-delivery roles/infrastructure; workforce capacity/implementation support	User participation	Role-perception evidence informed positioning and role domains, not effectiveness.
Rouch et al. (2022) [45]	Policy recognition; financing/coverage; referral/coordination; workforce/implementation; governance/collaboration	Entry into primary-care teams, team navigation, reimbursement and expansion barriers	Qualitative experience findings	Policy recognition/institutional positioning; financing/coverage; referral/coordination; workforce capacity/implementation support	None	Team navigation content was distributed to referral/coordination and implementation.
Missen et al. (2021) [46]	Service delivery; workforce/implementation; access/equity; system outcomes	Basic Assessment Model workflow; median wait 105 to 80 days	Uncontrolled pre/post service data	Service-delivery roles/infrastructure; workforce capacity/implementation support	Accessibility	Waiting-time change retained as direct quantitative outcome; causal attribution excluded.
Smith & Gallego (2021) [47]	Service delivery; access/equity; system outcomes	Outreach, attendance and transport/health-literacy barriers	Mixed-method service data/caregiver survey	Service-delivery roles/infrastructure	Accessibility; equity; user participation	Access barriers were treated as cross-cutting because they affected service reach.
Raymond et al. (2020) [48]	Referral/coordination; access/equity; governance/collaboration	Stakeholder differences in referral-prioritization rationales	Qualitative stakeholder perspectives	Referral/coordination	Equity; user participation	Safety, independence, and participation priorities were retained as cross-cutting considerations.
Bolt et al. (2019) [31]	Policy recognition; financing/coverage; service delivery; governance/collaboration	Cross-country service/funding organization plus professional recommendations	Survey separated from normative position statements	Policy recognition/institutional positioning; financing/coverage; service-delivery roles/infrastructure	None	An empirical survey informed the framework; professional recommendations were restricted in the sensitivity analysis.
Dickson & Toto (2018) [49]	Referral/coordination; service delivery; workforce/implementation; system outcomes	Feasibility of embedding OT in aging-in-place care coordination	Single-group feasibility findings	Referral/coordination; service-delivery roles/infrastructure; workforce capacity/implementation support	None	Feasibility was retained as an implementation observation, not as evidence of downstream effectiveness.
Friedman & VanPuymbrouck (2018) [50]	Policy recognition; financing/coverage; service delivery	Medicaid waiver coverage, projected utilization, expenditure, reimbursement, and service definitions	Document/quantitative descriptive analysis	Policy recognition/institutional positioning; financing/coverage; service-delivery roles/infrastructure	Equity in coverage variation	Coverage/reimbursement was a direct system arrangement; projected utilization was not treated as causal outcome evidence.
Dahl-Popolizio et al. (2017) [51]	Policy recognition; financing/coverage; service delivery; workforce/implementation	Receptiveness to OT on primary-care teams and perceived funding/role barriers	Descriptive survey perceptions	Policy recognition/institutional positioning; financing/coverage; service-delivery roles/infrastructure; workforce capacity/implementation support	None	Perceived barriers informed implementation conditions, not prevalence estimates.
Koverman et al. (2017) [52]	Financing/coverage; referral/coordination; service delivery; workforce/implementation	Logic model, EMR, scheduling, referral and reimbursement processes	Structured implementation observations	Financing/coverage; referral/coordination; service-delivery roles/infrastructure; workforce capacity/implementation support	None	Only traceable operational elements were coded; effectiveness/cost-saving claims excluded.
Raymond et al. (2016) [53]	Access/equity; service delivery; system outcomes	Waiting-list management strategies and association with longest waiting time	Cross-sectional program survey	Service-delivery roles/infrastructure	Accessibility; equity	Association retained with very-low GRADE certainty; no causal management effect inferred.
Wilberforce et al. (2016) [54]	Service delivery; workforce/implementation	Distribution of practitioner time across direct care, casework and team/service activity	Cross-sectional time-use data	Service-delivery roles/infrastructure; workforce capacity/implementation support	None	Time allocation was workforce/service-organisation evidence, not an outcome measure.
Donnelly et al. (2013) [55]	Policy recognition; referral/coordination; workforce/implementation; governance/collaboration	Communication, trust, role understanding, co-location, EMR and team meetings	Multiple-case qualitative findings	Policy recognition/institutional positioning; referral/coordination; workforce capacity/implementation support; service-delivery roles/infrastructure	None	Co-location/EMR mapped to infrastructure; communication/trust/team processes to coordination/implementation.
Raymond et al. (2013) [56]	Referral/coordination; access/equity; system outcomes	Referral-prioritisation criteria and differences in waiting time by priority	Program survey/content analysis	Referral/coordination	Accessibility; equity	Priority criteria were direct referral evidence; waiting differences informed access; companion publication controlled in sensitivity analysis.
London-Willis et al. (2012) [57]	Policy recognition; service delivery; workforce/implementation; governance/collaboration	Personalisation-related changes in OT role, identity, opportunities and threats	Qualitative perceptions	Policy recognition/institutional positioning; service-delivery roles/infrastructure; workforce capacity/implementation support	User participation context	Role identity/organisational change were retained as positioning/implementation evidence, not causal service effects.
Carrier et al. (2010) [58]	Access/equity; governance/collaboration; financing/resource allocation; referral/prioritisation	Access, prioritization, waiting, resource allocation, distributive justice and service quality	Legal/ethical/clinical analysis	Financing/coverage; referral/coordination	Accessibility; equity	Normative/legal analysis interpreted access/allocation principles, not service effects.
Quick et al. (2010) [59]	Financing/coverage; service delivery; workforce/implementation	Scope of practice, health promotion, funding/resource barriers and workforce characteristics	Descriptive survey	Financing/coverage; service-delivery roles/infrastructure; workforce capacity/implementation support	None	Scope/resource constraints informed service/workforce domains; no inference of effectiveness.

EMR, electronic medical record; GRADE, Grading of Recommendations Assessment, Development and Evaluation; OT, occupational therapy.

## Appendix E. Final Item-Level Design-Matched Critical-Appraisal Results

**Table:**

Study	Appraisal Tool	Final Item-Level Judgments	Key Limitation
Freeman et al. (2025) [12]	JBI analytical cross-sectional	Selection U; setting Y; exposure/system measurement U; confounding identification U; confounding control U; outcome measurement U; statistical inference Y; transferability U	Member-organization self-report and representativeness/measurement limitations.
Nash et al. (2025) [41]	MMAT mixed methods	S1 Y; S2 Y; M1 Y; M2 CT; M3 CT; M4 CT; M5 CT	Convenience/snowball sampling, very small interview sample, and limited integration.
Evans et al. (2023) [42]	MMAT mixed methods	S1 Y; S2 Y; M1 Y; M2 Y; M3 Y; M4 CT; M5 CT	Convenience sampling and self-developed survey; geographic overrepresentation.
Dibsdall (2022) [43]	JBI qualitative	Q1 Y; Q2 Y; Q3 Y; Q4 Y; Q5 Y; Q6 U; Q7 U; Q8 Y; Q9 Y; Q10 Y	Researcher positioning/influence incompletely reported.
Lim et al. (2022) [44]	QuADS	Q1–Q13: 3,3,3,3,2,3,3,3,3,2,3,2,2 (35/39; descriptive total only)	Purposive Q-sample/P-sample, non-loading participants, and limited reflexivity.
Rouch et al. (2022) [45]	JBI qualitative	Q1 U; Q2 Y; Q3 Y; Q4 Y; Q5 Y; Q6 Y; Q7 U; Q8 Y; Q9 Y; Q10 Y	Small sample; single PI conducted interviews and primary analysis.
Missen et al. (2021) [46]	JBI quasi-experimental	Temporal Y; comparable groups U; similar care N; control N; repeated measures Y; follow-up Y; same outcome measurement Y; reliable measurement Y; analysis Y	No concurrent control and staffing changes during implementation.
Smith & Gallego (2021) [47]	MMAT mixed methods	S1 Y; S2 CT; M1 Y; M2 CT; M3 CT; M4 CT; M5 N	Small parent/carer sample and potential selection bias; limited integration.
Raymond et al. (2020) [48]	JBI qualitative	Q1 U; Q2 Y; Q3 Y; Q4 Y; Q5 Y; Q6 Y; Q7 Y; Q8 Y; Q9 Y; Q10 Y	Scenario-based data and context-specific transferability.
Bolt et al. (2019) [31]	MMAT quantitative descriptive + JBI expert opinion	Survey: sampling CT; representativeness CT; measurement CT; non-response CT; analysis Y. Position: expertise Y; focus Y; logic Y; literature Y; alternatives U; bias U	Survey reporting is limited, and professional advocacy is mixed with empirical survey results.
Dickson & Toto (2018) [49]	JBI quasi-experimental	Temporal Y; comparable groups N; similar care N; control N; repeated measures N; follow-up U; same outcome measurement Y; reliable measurement Y; analysis Y	Small selected single group without comparator; short follow-up.
Friedman & VanPuymbrouck (2018) [50]	MMAT mixed methods	S1 Y; S2 Y; M1 Y; M2 Y; M3 Y; M4 CT; M5 CT	Projected rather than observed utilisation/expenditure; qualitative coding limitations.
Dahl-Popolizio et al. (2017) [51]	MMAT quantitative descriptive	Sampling N; representativeness N; measurement CT; non-response N; analysis Y	Convenience/snowball sampling, unknown denominator, unvalidated instrument, and priming.
Koverman et al. (2017) [52]	QuADS	Q1–Q13: 3,3,3,2,1,1,1,2,1,1,1,2,1 (22/39; descriptive total only)	Sampling, systematic data collection, analysis, ethics, and audit trail were incompletely reported.
Raymond et al. (2016) [53]	JBI analytical cross-sectional	Selection Y; setting Y; exposure/system measurement Y; confounding identification U; control U; outcome measurement U; inference Y; transferability U	Cross-sectional design, proxy waiting-list outcome, and residual confounding.
Wilberforce et al. (2016) [54]	JBI analytical cross-sectional	Selection Y; setting Y; exposure/system measurement U; confounding identification U; control U; outcome measurement U; inference Y; transferability U	Self-report diary tool with incomplete measurement-property evidence; single trust.
Donnelly et al. (2013) [55]	JBI qualitative	Q1 U; Q2 Y; Q3 Y; Q4 Y; Q5 Y; Q6 U; Q7 U; Q8 Y; Q9 Y; Q10 Y	Researcher positioning and influence were incompletely reported; four cases.
Raymond et al. (2013) [56]	JBI analytical cross-sectional	Selection Y; setting Y; exposure/system measurement Y; confounding identification U; control U; outcome measurement Y; inference Y; transferability U	Regional confounding and the same underlying Quebec program dataset as Raymond (2016). [53]
London-Willis et al. (2012) [57]	JBI qualitative	Q1 Y; Q2 Y; Q3 Y; Q4 Y; Q5 Y; Q6 Y; Q7 U; Q8 Y; Q9 Y; Q10 Y	Six early volunteers and no explicit saturation report.
Carrier et al. (2010) [58]	QuADS	Q1–Q13: 3,3,2,3,2,2,2,2,1,2,3,1,2 (28/39; descriptive total only)	Document search/selection flow, dual review, and audit trail incompletely reported.
Quick et al. (2010) [59]	MMAT quantitative descriptive	Sampling CT; representativeness CT; measurement CT; non-response CT; analysis Y	Population denominator, non-response bias, and instrument validation were incompletely reported.

CT, cannot tell; JBI, Joanna Briggs Institute; M1–M5, MMAT mixed-methods criteria 1–5; MMAT, Mixed Methods Appraisal Tool; N, no; PI, principal investigator; Q1–Q13, appraisal items/questions 1–13; QuADS, Quality Assessment for Diverse Studies; S1–S2, MMAT screening questions 1–2; U, unclear; Y, yes.

## Appendix F. Detailed Sensitivity-Analysis Rules, Results, and Interpretation Changes

**Table:**

Sensitivity Analysis	Rule	Result	What Changed	Manuscript Interpretation
S1. Exclude expert opinion and author-proposed recommendations	Remove professional-position statements and other normative recommendations; retain empirical research findings.	Five organizing domains remain identifiable.	Professional advocacy is not necessary to sustain the core framework.	Policy recommendations should be phrased as considerations, not observed evidence.
S2. Direct findings only	Retain findings directly documented or measured in policy documents, service records, surveys, time-use data, and operational reports.	Financing, referral, access, service organization, and workforce patterns remain.	Many role-expansion and broader prevention claims weaken.	Use direct evidence for descriptive system claims; avoid broad role claims.
S3. Remove outcomes not directly measured	Exclude hospitalization, institutionalization, cost-saving, and long-term-care demand claims unless directly measured.	Core policy/system domains unchanged.	RQ4 narrows to waiting time, feasibility, access, expenditure structures, and service activity.	Describe downstream outcomes as future hypotheses only.
S4. Control companion publications	Treat Raymond (2013) [56] and (2016) [53] as two reports from a single Quebec dataset.	Referral/access theme remains, but the evidence count is reduced by one unique dataset.	Prevents double-counting of sample and context.	Report 21 reports and 20 unique studies/datasets, if no other overlaps exist.
S5. Restrict Koverman to traceable operational data	Retain EMR, scheduling, billing, and implemented pilot facts; remove broad success and referral-effect claims.	The service infrastructure and implementation theme remain.	Claims about increased referrals and provider acceptance weaken.	Use Koverman only as a structured implementation report.
S6. Restrict uncontrolled studies to feasibility/local change	For Missen and Dickson, retain only feasibility and observed local change.	Access and implementation domains remain.	No causal effect conclusions remain.	Use ‘following implementation’ and ‘feasible’, not ‘caused’ or ‘effective’.

EMR, electronic medical record; RQ4, research question 4; S1–S6, sensitivity analyses 1–6.

## Appendix G. Full GRADE-CERQual Evidence Profile for Qualitative Synthesis Findings

**Table:**

Qualitative Review Finding	Contributing Qualitative Evidence	Methodological Limitations	Coherence	Adequacy	Relevance	Overall Confidence	Explanation
Role clarity and team understanding influence whether OT is integrated into routine team practice.	Donnelly (2013) [55]; Rouch (2022) [45]; Dibsdall (2022) [43]; London-Willis (2012) [57]	Moderate concerns	Minor concerns	Moderate concerns	Minor concerns	Moderate	Consistent pattern across primary care, reablement, and social care, but limited by small numbers of settings and incomplete reflexivity in some studies.
Co-location, shared records, team meetings, and managerial support facilitate organizational integration.	Donnelly (2013) [55]; Dibsdall (2022) [43]; Rouch (2022) [45]	Minor to moderate concerns	Minor concerns	Moderate concerns	Minor concerns	Moderate	Clear fit between data and findings across three qualitative studies, although the number of data-rich settings was limited.
Reimbursement and billing constraints impede the integration of OT into U.S. primary care.	Rouch (2022) [45]; qualitative component of Nash (2025) [41]	Moderate concerns	Minor concerns	Serious concerns	Moderate concerns	Low	The finding is coherent but supported by a few data-rich qualitative sources and is mainly relevant to the U.S. financing context.
Service context and workforce capacity shape how OTs enact their roles.	Dibsdall (2022) [43]; Donnelly (2013) [55]; Rouch (2022) [45]; London-Willis (2012) [57]	Moderate concerns	Minor concerns	Moderate concerns	Minor concerns	Moderate	Recurring across several service models, with some limitations in sampling, reflexivity, and contextual breadth.
Service users and professionals may differ in referral-priority and fairness judgments.	Raymond (2020) [48]	Minor to moderate concerns	Minor concerns	Serious concerns	Moderate concerns	Low	Rich within-study comparison, but based on one Canadian study using hypothetical scenarios.
Social-care or reablement redesign may create tension between expanded system roles and occupation-focused practice.	Dibsdall (2022) [43]; London-Willis (2012) [57]	Moderate concerns	Minor concerns	Serious concerns	Moderate concerns	Low	Coherent across two UK studies, but limited in quantity and jurisdictional relevance.

GRADE-CERQual, Grading of Recommendations Assessment, Development and Evaluation–Confidence in the Evidence from Reviews of Qualitative Research; OT, occupational therapy; OTs, occupational therapists; U.S., United States; UK, United Kingdom.

## Appendix H. Full GRADE Certainty Assessment for Eligible Quantitative Outcomes

**Table:**

Quantitative Outcome	Study/Body of Evidence	Starting Level	Risk of Bias	Inconsistency	Indirectness	Imprecision/Publication Bias	Final Certainty and Interpretation
Waiting-list time following introduction of a rural assessment model	Missen et al. (2021) [46]; one uncontrolled before–after service evaluation	Low (observational/non-randomized)	Serious: no concurrent control and staffing changes	Not assessable: one study	Not serious for the local wait-list outcome	Serious imprecision; publication bias undetectable	Very low. The observed local reduction cannot be attributed causally to the model.
Association between waiting-list management practices and longest waiting time	Raymond et al. (2016) [53]; 55 Quebec programs	Low (observational cross-sectional)	Serious: cross-sectional design and residual confounding	Not assessable: one study	Serious: the longest waiting person is a proxy for overall access	Imprecision not fully quantifiable; publication bias undetectable	Very low. Supports an observed association only, not a causal management effect.
Feasibility of integrating OT into a care-coordination program	Dickson and Toto (2018) [49]; one small single-group feasibility study	Low (observational/non-randomized)	Serious: selected sample, no comparator, short follow-up	Not assessable: one study	Not serious for feasibility in the studied program; serious for broader effects	Serious imprecision; publication bias undetectable	Very low. Supports feasibility under study conditions only.

GRADE, Grading of Recommendations Assessment, Development and Evaluation; OT, occupational therapy.
